# A Circular Touch Mode Capacitive Rainfall Sensor: Analytical Solution and Numerical Design and Calibration

**DOI:** 10.3390/s24196291

**Published:** 2024-09-28

**Authors:** Xiao-Ting He, Jun-Song Ran, Ji Wu, Fei-Yan Li, Jun-Yi Sun

**Affiliations:** 1 School of Civil Engineering, Chongqing University, Chongqing 400045, China; 202316131371@stu.cqu.edu.cn (J.-S.R.); 202116131099t@cqu.edu.cn (J.W.); 202116131224t@cqu.edu.cn (F.-Y.L.); sunjunyi@cqu.edu.cn (J.-Y.S.); 2 Key Laboratory of New Technology for Construction of Cities in Mountain Area (Chongqing University), Ministry of Education, Chongqing 400045, China

**Keywords:** capacitive sensor, rainfall measurement, large deflection, analytical solution, numerical calibration

## Abstract

A circular capacitive rainfall sensor can operate from non-touch mode to touch mode; that is, under the action of enough rainwater, its movable electrode plate can form a circular contact area with its fixed electrode plate. Therefore, the weight of rainwater is borne by only its movable electrode plate in non-touch mode operation but by both its movable and fixed electrode plates in touch mode operation, and the total capacitance of its touch mode operation is much larger than that of its non-touch mode operation. Essential to its numerical design and calibration is the ability to predict the deflection shape of its moveable electrode plate to determine its total capacitance. This requires the analytical solution to the fluid–structure interaction problem of its movable electrode plate under rainwater. In our previous work, only the analytical solution for the fluid–structure interaction problem before its movable electrode plate touches its fixed electrode plate was obtained, and how to numerically design and calibrate a circular non-touch mode capacitive rainfall sensor was illustrated. In this paper, the analytical solution for the fluid–structure interaction problem after its movable electrode plate touches its fixed electrode plate is obtained, and how to numerically design and calibrate a circular touch mode capacitive rainfall sensor is illustrated for the first time. The numerical results show that the total capacitance and rainwater volume when the circular capacitive rainfall sensor operates in touch mode is indeed much larger than that when the same circular capacitive rainfall sensor operates in non-touch mode, and that the average increase in the maximum membrane stress per unit rainwater volume when the circular capacitive rainfall sensor operates in touch mode can be about 20 times smaller than that when the same circular capacitive rainfall sensor operates in non-touch mode. This is where the circular touch mode capacitive rainfall sensor excels.

## 1. Introduction

A capacitive-type sensor relies mainly on a variable capacitor to operate, and generates capacitance as an output electrical signal in response to the input non-electrical signal [1,2,3,4,5]. The variable capacitor consists of a pair of electrode plates (which are usually coated by a thin layer of insulator and initially parallel to each other) and a medium filled in the gap between the two electrode plates, and is classified according to the following conditions: ① the parallel distance between the two initially parallel electrode plates is kept constant, while the permittivity of the medium between the two electrode plates is changed (a different medium is used) [6,7,8,9,10]; ② the parallel distance between the two initially parallel electrode plates and the permittivity of the medium between the two electrode plates are all kept constant, while one of the two initially parallel electrode plates is rotated by an angle with respect to the other to change the area of mutual projection between the two electrode plates [11,12,13,14,15]; ③ one of the two initially parallel electrode plates is moved in parallel with respect to the other to change the parallel distance between the two electrode plates, while the permittivity of the medium between the two electrode plates is kept constant (the same medium, such as air, is used before and after the parallel movement) [16,17,18,19,20]; and ④ one of the two initially parallel electrode plates is moved in a non-parallel manner with respect to the other to change the spatial geometry between the two electrode plates, while the permittivity of the medium between the two electrode plates is kept constant (the same medium, such as air, is used before and after the non-parallel movement) [21,22,23,24,25].

The above first type of variable capacitor can be incorporated into the development of a measurement system based on the change in permittivity, such as a capacitive moisture content sensor used for measuring the water content of the medium such as construction river sand or grain flour [26,27,28,29,30]. The above second type of variable capacitor can be incorporated into the development of a measurement system for the angle of rotation, such as a capacitive angular displacement sensor (an angular encoder) used for measuring the angle of rotation between a rotating part (the rotor) and a non-rotating part (the stator) [31,32,33,34,35]. The above third type of variable capacitor can be incorporated into the development of a measurement system for parallel movement, such as a capacitive displacement sensor used for measuring the force pushing an object in parallel [36,37,38,39,40]. The above fourth type of variable capacitor can be incorporated into the development of such capacitive pressure sensors [41,42,43,44,45]. Obviously, the above first three types are three parallel-plate variable capacitors, while the above fourth type is a non-parallel-plate variable capacitor. However, it is well-known that the calculation of the capacitance of a parallel-plate capacitor is very easy, while that of a non-parallel-plate capacitor is often very difficult (because it requires the spatial geometry of the movable electrode plate of the non-parallel-plate variable capacitor, which is often very difficult to obtain). In particular, a non-parallel-plate variable capacitor may operate in either the non-touch mode wherein the movable electrode plate does not come into contact with the unmovable electrode plate, or the touch mode wherein the movable electrode plate comes into contact with the unmovable electrode plate, and the spatial geometry of the movable electrode plate under the touch mode is often more difficult to obtain than that under the non-touch mode, but, in applications, touch mode operation can offer a better performance than non-touch mode operation [46,47,48,49,50].

In previous studies [51,52], we suggested the use of the non-parallel-plate variable capacitor for developing a new rain gauge, an instrument for measuring the amount of rain that falls. The currently available rain gauges, in addition to manual rain gauges, mainly include weighing rain gauges [53,54] and tipping-bucket rain gauges [55,56,57,58,59], where the tipping-bucket rain gauges are the most popular due to their simplicity and robustness. However, the tipping-bucket and weighing rain gauges usually need very troublesome static or dynamic calibrations in the laboratory and in the field [60,61,62,63], and the rainfall measurement errors caused by calibration errors may be as high as 40% [64,65,66,67,68]. Unlike the tipping-bucket or weighing rain gauges, the new rain gauge proposed in [51,52] does not need to calibrate the volumetric metering of rainwater, thus avoiding the rainfall measurement errors caused by calibration errors. However, due to the difficulty in obtaining the spatial geometry of the unmovable electrode plate under the touch mode, only the circular capacitive rainfall sensor operating in the non-touch mode was theoretically studied in [51,52]. In view of the greater application potential of touch mode operation than that of non-touch mode operation, this paper presents a theoretical study on the circular capacitive rainfall sensor operating in the touch mode for the first time.

The paper is organized as follows: In order to accurately calculate the total capacitance of the circular capacitive rainfall sensor operating in the touch mode, the elastic behavior of the circular movable electrode plate of the variable capacitor of the circular touch mode capacitive rainfall sensor is analytically solved in Section 2.1. The input–output relationship of the circular touch mode capacitive rainfall sensor is accurately derived in Section 2.2. An example illustration of how to use the analytical solution and input–output relationship in Section 2 to numerically design and calibrate a circular touch mode capacitive rainfall sensor is given in Section 3.1. The effect of changing the design parameters of the circular touch mode capacitive rainfall sensor on its input–output relationship is given in Section 3.2. Concluding remarks are given in Section 4.

## 2. Materials and Methods

**Figure 1:**
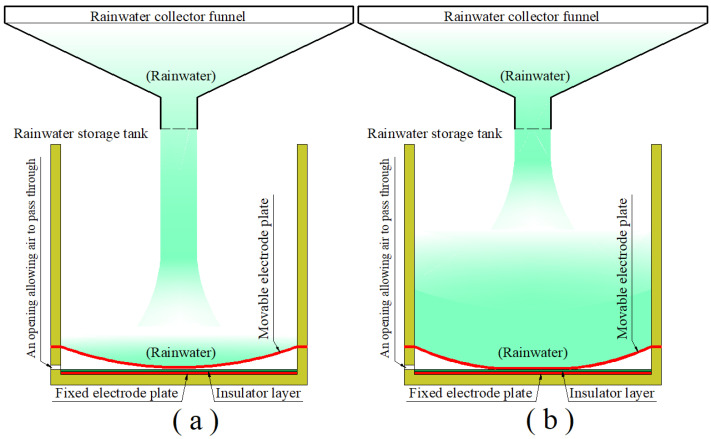
Schematic of a rainfall measurement system using a circular capacitive sensor operating in touch mode: (**a**) the occasion when the movable electrode plate is just in contact with the insulator layer; and (**b**) the case after contact between the movable electrode plate and the insulator layer.

The term “rainfall” refers to the total amount of rain that falls in a particular area in a particular amount of time, usually measured in millimeters. Usually, a funnel that is wide at the top and narrow at the bottom is placed outside to be used to collect the water that falls from the sky to the ground when it rains. The total volume of the rainwater that is collected over a given period of time divided by the top opening area of the rainwater collector funnel is the rainfall, as shown in Figure 1, where the rainwater collected is poured into a cylindrical storage tank through the bottom opening of the funnel to measure its total volume. The bottom of the cylindrical rainwater storage tank is made of a circular conductive membrane, and, before the tank holds rainwater, the circular conductive membrane is initially flat, and, after the tank holds rainwater, it will elastically deflect axisymmetrically. Therefore, if the initially flat circular conductive membrane coaxially faces a circular conductive rigid thin plate coated by a thin layer of insulator, where the membrane and plate have the same radius and keep a parallel gap, then the circular conductive membrane and rigid thin plate can be used as a moveable electrode plate and a fixed electrode plate to form a non-parallel-plate variable capacitor, as shown in Figure 1.

Obviously, the axisymmetric non-parallel deflections of the moveable electrode plate (circular conductive membrane) toward the fixed electrode plate will increase with the increase in the amount of the rainwater collected in the storage tank, that is, with the increase in the volume of the rainwater in the storage tank. It is conceivable that, when the rainwater collected increases to a suitable volume, the moveable electrode plate (circular conductive membrane) will just touch the insulator layer on the fixed electrode plate, as shown in Figure 1a. Then, as the volume of the collected rainwater further increases, a circular contact area will be formed between the deflected moveable electrode plate (circular conductive membrane) and the insulator layer on the fixed electrode plate, as shown in Figure 1b. The elastic behavior of the moveable electrode plate (circular conductive membrane) under the action of the collected rainwater can be mechanically regarded as a plate/membrane contact problem of an initially flat, peripherally fixed circular membrane under the action of non-uniformly distributed transverse loads after the deflected moveable electrode plate (circular conductive membrane) touches the insulator layer on the fixed electrode plate, and as a large deflection problem of an initially flat, peripherally fixed circular membrane under the action of non-uniformly distributed transverse loads before the deflected moveable electrode plate touches the insulator layer. Only the former, the plate/membrane contact problem, needs to be dealt with here, while the latter, the large deflection problem, has been dealt with originally in [69] and then improved in [51,52].

In order to accurately calculate the capacitance of the non-parallel-plate variable capacitor operating in touch mode, it is necessary to be able to mathematically describe the spatial geometry of the deflected movable electrode plate (circular conductive membrane) touching the insulator layer on the fixed electrode plate, which requires the analytical solution to the plate/membrane contact problem between the deflected movable electrode plate (circular conductive membrane) and the insulator layer on the fixed electrode plate, which is also a key scientific problem to be solved in this study. The analytical difficulties are mainly caused by the interaction between the rainwater collected in the storage tank and the movable electrode plate (circular conductive membrane) that is subjected to the action of the rainwater collected, i.e., the so-called fluid–structure coupling problem. Obviously, as the amount of the rainwater collected in the storage tank increases, the circular contact area between the deflected moveable electrode plate (circular conductive membrane) and the insulator layer on the fixed electrode plate will increase, and the spatial geometry of the deflected movable electrode plate (circular conductive membrane) in the plate/membrane non-contact area will also change—see Figure 1b. That is to say, the rainwater in the storage tank, as external loads acting on the moveable electrode plate (circular conductive membrane), makes the moveable electrode plate (circular conductive membrane) axisymmetrically elastically deflect in a non-parallel manner, and the increasing rainwater in the storage tank constantly changes the geometrical shape of the deflected moveable electrode plate (circular conductive membrane). On the other hand, the geometrical shape of the deflected moveable electrode plate (circular conductive membrane) determines the distribution of the rainwater in the storage tank on the deflected moveable electrode plate (circular conductive membrane); that is, the constant change in the geometrical shape of the deflected moveable electrode plate (circular conductive membrane) continuously changes the distribution of the external loads acting on the moveable electrode plate (circular conductive membrane). Therefore, the increasing rainwater in the storage tank constantly changes the geometrical shape of the deflected moveable electrode plate (circular conductive membrane), and the constant change in the geometrical shape of the deflected moveable electrode plate (circular conductive membrane) will, in turn, continuously change the distribution of the external loads acting on the moveable electrode plate (circular conductive membrane), thus constituting a fluid–structure coupling problem.

Such a fluid–structure interaction has made it complicated enough to solve the large deflection problem analytically, let alone the analytical solution to the plate/membrane contact problem. Therefore, we first focus on overcoming the analytical difficulties of the plate/membrane contact problem (see Section 2.1), and then derive the input–output relationship of the circular touch mode capacitive rainfall sensor (see Section 2.2).

### 2.1. Analytical Solution to Elastic Behavior of Deflected Movable Electrode Plate in Touch Mode

**Figure 2:**
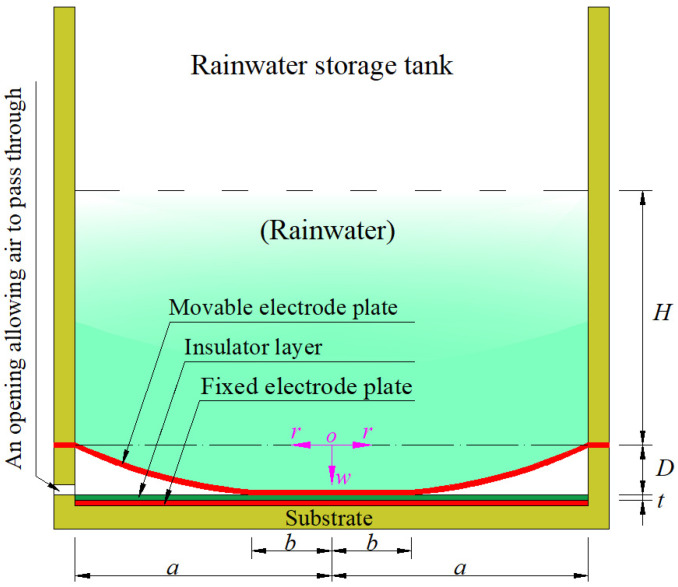
Structural parameters of a circular touch mode capacitive rainfall sensor.

Suppose that a circular capacitive rainfall sensor uses an initially flat circular conductive membrane with radius *a*, thickness *h*, Poisson’s ratio *ν*, and Young’s modulus of elasticity *E* as the movable electrode plate of its non-parallel-plate variable capacitor, and operates in touch mode when its cylindrical rainwater storage tank with inner radius *a* stores the rainwater with the height *H*, as shown in Figure 2, where the dash-dotted line represents the geometric middle plane of the initially flat moveable electrode plate (circular conductive membrane) that has not yet been subjected to the action of the rainwater and is peripherally fixed and tightly sealed on the wall of the cylindrical rainwater storage tank: *H* is the height from the geometric middle plane to the rainwater upper surface, *D* is the distance from the geometric middle plane to the upper surface of the insulator layer with thickness *t*, *b* is the contact radius between the deflected circular movable electrode plate (circular conductive membrane) and the insulator layer, and a cylindrical coordinate system (*r*, *φ*, *w*) is introduced, whose coordinate origin *o* is coincident with the centroid of the geometric middle plane, polar plane (*r*, *φ*) is located in the plane in which the geometric middle plane is located, and radial, circumferential, and transverse coordinates are *r*, *φ* and *w*, respectively; moreover, *w* denotes the deflection (transverse displacement) of the movable electrode plate (circular conductive membrane) under the action of the rainwater in the cylindrical rainwater storage tank. In addition, the fixed electrode plate is made of a circular conductive rigid thin plate that is coaxially parallel to the initially flat movable electrode plate (circular conductive membrane) and is fixed to a non-conductive substrate—see Figure 2.

The elastic behavior of the moveable electrode plate (circular conductive membrane) in contact with the insulator layer on the fixed electrode plate under the action of the rainwater in the rainwater storage tank can be mechanically simplified as a plate/membrane contact problem wherein an initially flat, peripherally fixed circular membrane comes into frictionless axisymmetric contact with a rigid thin plate under the action of non-uniformly distributed transverse loads. It can be seen from Figure 2 that the rainwater in the rainwater storage tank, as transverse loads acting on the circular membrane, is distributed axisymmetrically, although non-uniformly, on the deflected circular membrane. Therefore, the distribution of the rainwater in the rainwater storage tank is only related to the radial coordinate *r*, and has nothing to do with the circumferential coordinate *φ*.

**Figure 3:**
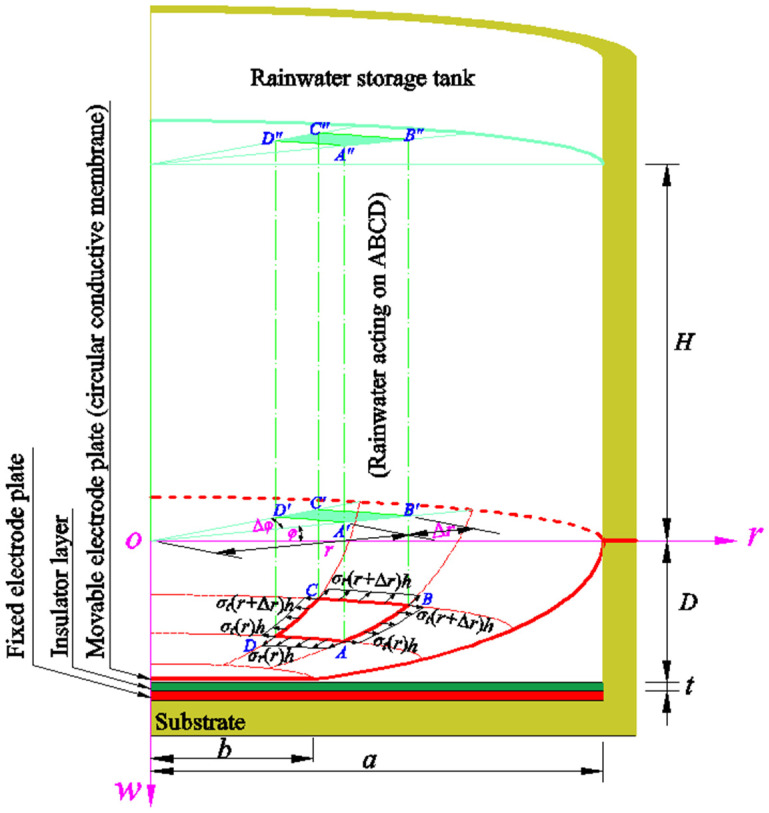
Half cross-sectional view of a circular touch mode capacitive rainfall sensor.

Let us first deal with the static problem of equilibrium of the axisymmetric deformation membrane in the plate/membrane non-contact annular region (*b* ≤ *r* ≤ *a*). If a radial coordinate increment Δ*r* and a circumferential coordinate increment Δ*φ* are taken in the polar plane (*r*, *φ*) to form a micro-area element A′B′C′D′, whose projection on the deflected circular membrane is ABCD, and whose projection on the surface of the rainwater in the rainwater storage tank is A′B′C′D′, then the transverse loads distributed on the micro-area element ABCD can be approximated by *ρg*[*H* + *w*(*r*)]Δ*φ*(2*r*Δ*r* + Δ*r*^2^)/2, as shown in Figure 3, where the coordinate of point A is (*r*, *φ*, *w*(*r*)), the coordinate of point B is (*r* + Δ*r*, *φ*, *w*(*r* + Δ*r*)), the coordinate of point C is (*r* + Δ*r*, *φ* + Δ*φ*, *w*(*r* + Δ*r*)), and the coordinate of point D is (*r*, *φ* + Δ*φ*, *w*(*r*)). In addition, the downward component of the radial force *r*Δ*φσ*_r_(*r*)*h* acting on the AD boundary of the micro-area element ABCD can be expressed as *r*Δ*φσ*_r_(*r*)*h*sin*θ*(*r*), and the upward component of the radial force (*r* + Δ*r*)Δ*φσ*_r_(*r* + Δ*r*)*h* acting on the BC boundary of the micro-area element ABCD can be expressed as (*r* + Δ*r*)Δ*φσ*_r_(*r* + Δ*r*)*h*sin*θ*(*r* + Δ*r*), where *θ*(*r*) is the slope angle at *r* and *θ*(*r* + Δ*r*) is the slope angle at *r* + Δ*r*—see Figure 3.

Therefore, the static equilibrium of the micro-area element ABCD in the direction perpendicular to the polar plane (*r*, *φ*), i.e., the so-called out-of-plane equilibrium, is governed by

(1) $$\frac{\Delta \varphi }{2}(2r\Delta r+\Delta r^{2})[H+w(r)]\rho g+r\Delta \varphi \sigma_{r}(r)h\sin\theta (r)-(r+\Delta r)\Delta \varphi \sigma_{r}(r+\Delta r)h\sin\theta (r+\Delta r)=0,$$

where *ρ* denotes the density of the rainwater collected and *g* denotes the acceleration of gravity. Let us expand *σ_r_*(*r* + Δ*r*) and sin*θ*(*r* + Δ*r*) into the Taylor series and ignore the higher-order terms therein

(2) $$\sigma_{r}(r+\Delta r)=\sigma_{r}(r)+\frac{d\sigma_{r}(r)}{dr}\Delta r+\frac{1}{2!}\frac{d^{2}\sigma_{r}(r)}{dr^{2}}{\left(\Delta r\right)}^{2}+\ldots \approx \sigma_{r}(r)+\frac{d\sigma_{r}(r)}{dr}\Delta r$$

and

(3) $$s\mathrm{in}\theta (r+\Delta r)=\sin\theta (r)+\frac{\mathrm{dsin}\theta (r)}{dr}\Delta r+\frac{1}{2!}\frac{d^{2}\sin\theta (r)}{dr^{2}}{\left(\Delta r\right)}^{2}+\ldots \approx \sin\theta (r)+\frac{\mathrm{dsin}\theta (r)}{dr}\Delta r.$$

Substituting Equations (2) and (3) into Equation (1), and, then, taking the limit as Δ*r* → 0 yield

(4) $$r[H+w(r)]\rho g-h\frac{d}{dr}[r\sigma_{r}(r)\sin\theta (r)]=0,$$

where *ρ* denotes the density of the rainwater collected and *g* denotes the acceleration of gravity. Therefore, if *w*(*r*) and *σ_r_*(*r*) are abbreviated to *w* and *σ_r_* and sin*θ*(*r*) is approximated by tan*θ*(*r*) = −d*w*/d*r*, then, from Equation (4), the out-of-plane equilibrium equation can finally be written as the following form

(5) $$r[H+w]\rho g+h\frac{d}{dr}[r\sigma_{r}\frac{dw}{dr}]=0.$$

In the direction parallel to the polar plane (*r*, *φ*), there is the circumferential force Δ*r*[*σ_t_*(*r*)*h*+ *σ_t_*(*r* + Δ*r*)*h*]sin(Δ*φ*/2) acting on the AB and CD boundaries of the micro-area element ABCD—see Figure 3. In addition, the horizontal component of the radial force *r*Δ*φσ_r_*(*r*)*h* acting on the AD boundary of the micro-area element ABCD can be expressed as *r*Δ*φσ_r_*(*r*)*h*cos*θ*(*r*), and the horizontal component of the radial force (*r* + Δ*r*)Δ*φσ_r_*(*r* + Δ*r*)*h* acting on the BC boundary of the micro-area element ABCD can be expressed as (*r* + Δ*r*)Δ*φσ_r_*(*r* + Δ*r*)*h*cos*θ*(*r* + Δ*r*). Therefore, the static equilibrium of the micro-area element ABCD in the direction parallel to the polar plane (*r*, *φ*), i.e., the so-called in-plane equilibrium, is governed by

(6) $$(r+\Delta r)\Delta \varphi \sigma_{r}(r+\Delta r)h\cos\theta (r+\Delta r)-r\Delta \varphi \sigma_{r}(r)h\cos\theta (r)-\Delta r[\sigma_{t}(r)h+\sigma_{t}(r+\Delta r)h]\sin\frac{\Delta \varphi }{2}=0.$$

Let us expand *σ_t_*(*r* + Δ*r*) and cos*θ*(*r* + Δ*r*) into the Taylor series and ignore the higher-order terms therein

(7) $$\sigma_{t}(r+\Delta r)=\sigma_{t}(r)+\frac{d\sigma_{t}(r)}{dr}\Delta r+\frac{1}{2!}\frac{d^{2}\sigma_{t}(r)}{dr^{2}}{\left(\Delta r\right)}^{2}+\ldots \approx \sigma_{t}(r)+\frac{d\sigma_{t}(r)}{dr}\Delta r$$

and

(8) $$\cos\theta (r+\Delta r)=\cos\theta (r)+\frac{d\cos\theta (r)}{dr}\Delta r+\frac{1}{2!}\frac{d^{2}\cos\theta (r)}{dr^{2}}{\left(\Delta r\right)}^{2}+\ldots \approx \cos\theta (r)+\frac{d\cos\theta (r)}{dr}\Delta r.$$

Substituting Equations (2), (7), and (8) into Equation (6), taking sin(Δ*φ*/2) ≈ Δ*φ*/2, and, then, taking the limit as Δ*r* → 0 and Δ*φ* → 0, considering sin*θ*(*r*) ≈ tan*θ*(*r*) used in the above out-of-plane equilibrium, and abbreviating *σ_r_*(*r*) and *σ_t_*(*r*) to *σ_r_* and *σ_t_*, the in-plane equilibrium equation can finally be written as the following form

(9) $$\frac{d}{dr}(r\sigma_{r})-\sigma_{t}=0.$$

**Figure 4:**
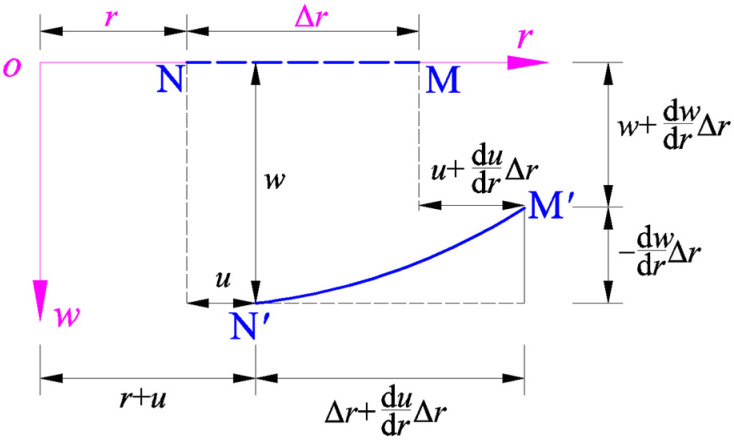
The geometric relationship between the micro radial straight line element $\bar{\mathrm{NM}}$ and the micro meridional curve element $\overset{⏜}{N^{\prime }M^{\prime }}$.

The geometric relationship between the radial strain and the radial and transverse displacements, i.e., the so-called radial geometric equation, can be formulated as follows: A micro straight line element $\bar{\mathrm{NM}}$ with length ∆*r* is assumed to be taken along the radial direction on the polar plane (*r*, *φ*), and, after deformation, it becomes a micro curve element $\overset{⏜}{N^{\prime }M^{\prime }}$ along the meridian direction of the deflected circular membrane, making the material particle N produce the radial displacement *u* and transverse displacement *w*, as shown in Figure 4.

Therefore, for the radial deformation from $\bar{\mathrm{NM}}$ to $\overset{⏜}{N^{\prime }M^{\prime }}$, the line strain in the radial direction may be written as

(10) $$e_{r}=\frac{\overset{⏜}{N^{\prime }M^{\prime }}-\bar{\mathrm{NM}}}{\bar{\mathrm{NM}}}=\frac{\left(\overset{⏜}{N^{\prime }M^{\prime }}+\bar{\mathrm{NM}})(\overset{⏜}{N^{\prime }M^{\prime }}-\bar{\mathrm{NM}}\right)}{(\overset{⏜}{N^{\prime }M^{\prime }}+\bar{\mathrm{NM}})\bar{\mathrm{NM}}}=\frac{{\left(\overset{⏜}{N^{\prime }M^{\prime }}\right)}^{2}-{\left(\bar{\mathrm{NM}}\right)}^{2}}{\overset{⏜}{N^{\prime }M^{\prime }}\bar{\mathrm{NM}}+\bar{\mathrm{NM}}\bar{\mathrm{NM}}}\approx \frac{{\left(\overset{⏜}{N^{\prime }M^{\prime }}\right)}^{2}-{\left(\bar{\mathrm{NM}}\right)}^{2}}{2{\left(\bar{\mathrm{NM}}\right)}^{2}}.$$

The length of the straight line $\bar{\mathrm{NM}}$ may be denoted by $L_{\bar{\mathrm{NM}}}=\Delta r$, while the length of the curve $\overset{⏜}{N^{\prime }M^{\prime }}$ may be approximated by

(11) $$L_{\overset{⏜}{N^{\prime }M^{\prime }}}\approx \sqrt{{\left(\Delta r+\frac{du}{dr}\Delta r\right)}^{2}+{\left(-\frac{dw}{dr}\Delta r\right)}^{2}}.$$

Therefore, the geometric relationship between the radial strain and the radial and transverse displacements, i.e., the radial geometric equation, may finally be written as

(12) $$e_{r}=\frac{L_{\overset{⏜}{N^{\prime }M^{\prime }}}^{2}-L_{\bar{\mathrm{NM}}}^{2}}{2L_{\bar{\mathrm{NM}}}^{2}}=\frac{{\left(\Delta r+\frac{du}{dr}\Delta r\right)}^{2}+{\left(-\frac{dw}{dr}\Delta r\right)}^{2}-\Delta r^{2}}{2\Delta r^{2}}=\frac{{\left(1+\frac{du}{dr}\right)}^{2}+{\left(-\frac{dw}{dr}\right)}^{2}-1}{2}\approx \frac{du}{dr}+\frac{1}{2}{\left(-\frac{dw}{dr}\right)}^{2}.$$

**Figure 5:**
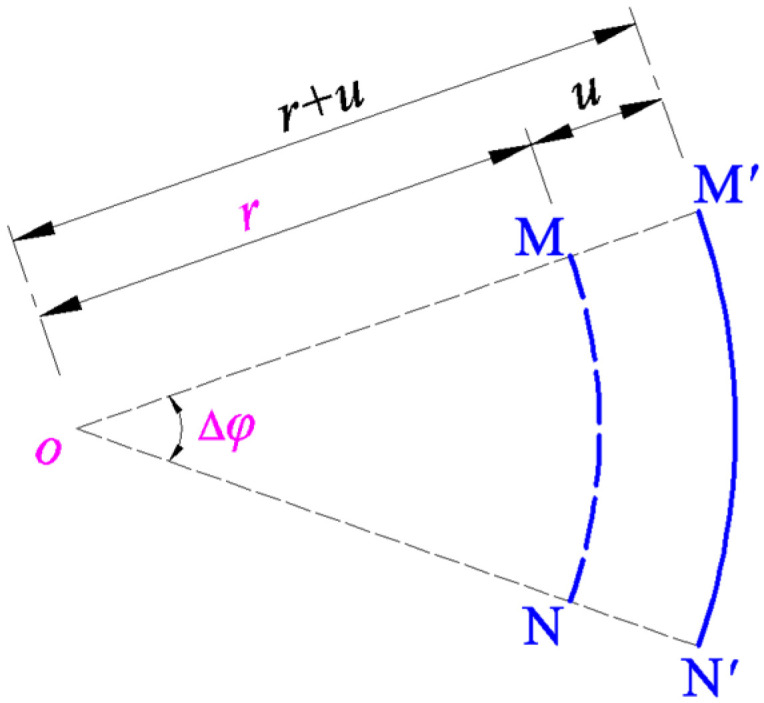
The geometric relationship between the circumferential curve micro elements $\overset{⏜}{\mathrm{NM}}$ and $\overset{⏜}{N\prime M\prime }$.

In addition, the geometric relationship between the circumferential strain and the radial displacement, i.e., the so-called circumferential geometric equation, can be formulated as follows: A micro curve element $\overset{⏜}{\mathrm{NM}}$ with radius *r* and central angle ∆*φ* is assumed to be taken along the circumferential direction on the polar plane (*r*, *φ*), and, after deformation, it becomes a micro curve element $\overset{⏜}{N^{\prime }M^{\prime }}$ along the circumferential direction of the deflected circular membrane, making the material particles N and M simultaneously produce the radial displacement *u*, as shown in Figure 5.

For the circumferential deformation from $\overset{⏜}{\mathrm{NM}}$ to $\overset{⏜}{N^{\prime }M^{\prime }}$, the line strain in the circumferential direction may be written as

(13) $$e_{t}=\frac{\overset{⏜}{N^{\prime }M^{\prime }}-\overset{⏜}{\mathrm{NM}}}{\overset{⏜}{\mathrm{NM}}}.$$

The lengths of the circumferential curve micro elements $\overset{⏜}{\mathrm{NM}}$ and $\overset{⏜}{N^{\prime }M^{\prime }}$ are, respectively,

(14) $$L_{\overset{⏜}{\mathrm{NM}}}=r\cdot \Delta \varphi$$

and

(15) $$L_{\overset{⏜}{N^{\prime }M^{\prime }}}=(r+u)\cdot \Delta \varphi .$$

Therefore, the geometric relationship between the circumferential strain and the radial displacement, i.e., the circumferential geometric equation, may finally be written as

(16) $$e_{t}=\frac{\overset{⏜}{N^{\prime }M^{\prime }}-\overset{⏜}{\mathrm{NM}}}{\overset{⏜}{\mathrm{NM}}}=\frac{(r+u)\cdot \Delta \varphi -r\cdot \Delta \varphi }{r\cdot \Delta \varphi }=\frac{u}{r}.$$

The physical relationships between stresses and strains, i.e., the so-called physical equations, are assumed to satisfy the generalized Hooke’s law, and are thus given by

(17a,b) $$\sigma_{r}=\frac{E}{1-\nu^{2}}(e_{r}+\nu e_{t})\mathrm{or}\;e_{r}=\frac{1}{E}(\sigma_{r}+\nu \sigma_{t})$$

and

(18a,b) $$\sigma_{t}=\frac{E}{1-\nu^{2}}(e_{t}+\nu e_{r})\mathrm{or}\;e_{t}=\frac{1}{E}(\sigma_{t}+\nu \sigma_{r}).$$

The general solutions of the six physical quantities radial stress *σ_r_*, circumferential stress *σ_t_*, radial strain *e_r_*, circumferential strain *e*_t_, radial displacement *u*, and transverse displacement (deflection) *w* can all be expressed as functions of the radial coordinate *r*, by solving the out-of-plane equilibrium equation, Equation (5), the in-plane equilibrium equation, Equation (9), the radial geometric equation, Equation (12), the circumferential geometric equation, Equation (16), and the physical equations, Equations (17a) and (18a) or Equations (17b) and (18b), simultaneously. The essentially arbitrary constants of these general solutions, also called undetermined constants, can be determined by the following boundary conditions at *r* = *a* and continuity conditions at *r* = *b*. The boundary conditions at *r* = *a* are

(19) w=0at r=a

and

(20) $$e_{t}=\frac{1}{E}(\sigma_{t}+\nu \sigma_{r})=0\mathrm{at}\;r=a.$$

The continuity conditions at *r* = *b* are

(21) w=Dat r=b,

(22) $$\frac{dw}{dr}=0\mathrm{at}\;r=b,$$

(23) $${\left(\sigma_{r}\right)}_{A}={\left(\sigma_{r}\right)}_{B}\mathrm{at}\;r=b$$

and

(24) $${\left(e_{t}\right)}_{A}={\left(e_{t}\right)}_{B}\mathrm{at}\;r=b,$$

where the two symbols ()*_A_* and ()*_B_* represent the two sides of the interconnecting circle of *r* = *b*, and the subscript *A* refers to the side of plate/membrane non-contact annular region (*b* ≤ *r* ≤ *a*), while the subscript *B* refers to the side of the plate/membrane contact circular region (0 ≤ *r* ≤ *b*).

Now, let us deal with the static problem of equilibrium of the axisymmetric deformation membrane in the plate/membrane contact circular region (0 ≤ *r* ≤ *b*). Obviously, d*w*/d*r* ≡ 0 for 0 ≤ *r* ≤ *b*, due to the fact that the membrane is always flat in the plate/membrane contact cirular region of 0 ≤ *r* ≤ *b*. Therefore, substituting d*w*/d*r* = 0 into Equation (12) yields

(25) $$e_{r}=\frac{du}{dr}.$$

Substituting Equations (16) and (25) into Equations (17a) and (18a) yields

(26) $$\sigma_{r}=\frac{E}{1-\nu^{2}}(\frac{du}{dr}+\nu \frac{u}{r})$$

and

(27) $$\sigma_{t}=\frac{E}{1-\nu^{2}}(\frac{u}{r}+\nu \frac{du}{dr}).$$

Substituting Equations (26) and (27) into Equation (9), we obtain

(28) $$r\frac{d^{2}u}{dr^{2}}+\frac{du}{dr}-\frac{u}{r}=0.$$

Since Equation (28) satisfies the form of the Euler equation, therefore, the general solution of Equation (28) may be written as

(29) $$u(r)=C_{1}r+\frac{C_{2}}{r},$$

where *C*_1_ and *C*_2_ are two unknown constants. It is not difficult to understand that *C*_2_ has to be equal to zero due to *u*(*r*) = 0 at *r* = 0. On the other hand, since *u*(*r*) = (*u*(*b*))*_B_* at *r = b*, then *C*_1_= (*u*(*b*))*_B_*/*b*, and, after (*u*(*b*))*_B_* is abbreviated to *u_B_*, *C*_1_= *u_B_*/*b*. Therefore, from Equation (29), the solution of Equation (28) may finally be written as

(30) $$u(r)=\frac{u_{B}}{b}r.$$

Substituting Equation (30) into Equations (26) and (27) and Equations (25) and (16) yields

(31) $$\sigma_{r}=\sigma_{t}=\frac{E}{1-\nu }\frac{u_{B}}{b}$$

and

(32) $$e_{r}=e_{t}=\frac{u_{B}}{b}.$$

Equations (31) and (32) suggest that the stresses and strains are uniformly distributed in the plate/membrane contact circular region of 0 ≤ *r* ≤ *b*. It is important to note that *u_B_* is a parameter that can only be determined after the static problem of equilibrium in the non-contact annular region of *b* ≤ *r* ≤ *a* has been solved analytically.

Now, let us continue the analytical solution to the static problem of equilibrium in the non-contact annular region of *b* ≤ *r* ≤ *a*. Eliminating *e_r_* and *e_t_* from Equations (12) and (16) and Equations (17a) and (18a) yields

(33) $$\sigma_{r}=\frac{E}{1-\nu^{2}}\left\{\frac{du}{dr}+\frac{1}{2}{\left(\frac{dw}{dr}\right)}^{2}+\nu \frac{u}{r}\right\}$$

and

(34) $$\sigma_{t}=\frac{E}{1-\nu^{2}}\left\{\frac{u}{r}+\nu [\frac{du}{dr}+\frac{1}{2}{\left(\frac{dw}{dr}\right)}^{2}]\right\}.$$

It is found from Equations (16) and (18b) that

(35) $$\frac{u}{r}=\frac{1}{E}(\sigma_{t}-\nu \sigma_{r}).$$

Substituting the *u* in Equation (35) into Equation (33) yields

(36) $$\frac{1}{E}\sigma_{r}-\frac{\nu }{E}\sigma_{t}-\frac{d}{dr}[\frac{1}{E}(r\sigma_{t}-\nu r\sigma_{r})]-\frac{1}{2}{\left(\frac{dw}{dr}\right)}^{2}=0.$$

Equations (36), (9), and (5) are three equations for solving the radial stress *σ_r_*, circumferential stress *σ_t_*, and transverse displacement (deflection) *w*. As long as the solutions of *σ_r_*, *σ_t_*, and *w* can be obtained, then the solutions of the radial strain *e_r_*, circumferential strain *e*_t_, and radial displacement *u* are easily obtained. To this end, let us introduce the following dimensionless variables

(37) $$W=\frac{w}{a},\;S_{r}=\frac{\sigma_{r}}{E},\;S_{t}=\frac{\sigma_{t}}{E},\;x=\frac{r}{a},\;\alpha =\frac{b}{a},H_{0}=\frac{H}{a},G=\frac{\rho ga^{2}}{Eh},$$

and transform Equations (5), (9), and (36) into

(38) $$S_{r}(-\frac{dW}{dx})+x\frac{dS_{r}}{dx}(-\frac{dW}{dx})-xS_{r}\frac{d^{2}W}{dx^{2}}-x[H_{0}+W(x)]G=0,$$

(39) $$\frac{d}{dx}(xS_{r})-S_{t}=0,$$

(40) $$S_{r}-\nu S_{t}-\frac{d}{dx}(xS_{t}-\nu xS_{r})-\frac{1}{2}{\left(\frac{dW}{dx}\right)}^{2}=0.$$

After considering Equations (31), (32), and (37), Equations (19)–(24) can be transformed into

(41) W=0at x=1,

(42) $$S_{t}-\nu S_{r}=0\mathrm{at}\;x=1,$$

(43) $$W=\frac{g}{a}\mathrm{at}\;x=\alpha ,$$

(44) $$\frac{dW}{dx}=0\mathrm{at}\;x=\alpha ,$$

(45) $${\left(S_{r}\right)}_{A}=\frac{1}{1-\nu }\frac{u_{B}}{b}\mathrm{at}\;x=\alpha$$

and

(46) $${\left(S_{t}-\nu S_{r}\right)}_{A}=\frac{u_{B}}{b}\mathrm{at}\;x=\alpha .$$

Since *S_r_*(*x*), *S_t_*(*x*), and *W*(*x*) are all finite in *α* ≤ *x* ≤ 1 (i.e., in *b* ≤ *r* ≤ *a*), they can be expanded into power series of *x* − *β* (*β* = (1 + *α*)/2)

(47) $$S_{r}=\sum_{i=0}^{\infty }b_{i}{\left(x-\beta \right)}^{i},$$

(48) $$S_{t}=\sum_{i=0}^{\infty }c_{i}{\left(x-\beta \right)}^{i}$$

and

(49) $$W=\sum_{i=0}^{\infty }d_{i}{\left(x-\beta \right)}^{i}.$$

Substitute Equations (47)–(49) into Equations (38)–(40), and combine like terms into one term according to the *x* with the same power, and, then, let all the sums of coefficient of the like terms be equal to zero, which will result in a system of infinitely many equations for the power series coefficients *b_i_*, *c_i_,* and *d_i_* (*i* = 0,1, 2, 3, …). The recursive relation for the power series coefficients *b_i_*, *c_i_,* and *d_i_* (*i* = 0,1, 2, 3, …) can be obtained by solving this system of equations, which are listed in Appendix A, where the coefficients *b_i_* and *c_i_* (*i* = 1, 2, 3, …) and the coefficients *d_i_* (*i* = 2, 3, 4, …) are expressed as the polynomials with regard to the first few coefficients *b*_0_, *c*_0_, *d*_0_, *d*_1_, and *β* (*β* = (1 + *α*)/2 and *α* = *b*/*a*).

The first few coefficients *b*_0_, *c*_0_, *d*_0_, *d*_1_, and *β* (or *α* or *b*) are called undetermined constants, which can be determined by using the boundary conditions, Equations (41) and (42), and continuity conditions, Equations (43)–(46). From Equation (49), Equations (41), (43), and (44) give

(50) $$\sum_{i=0}^{\infty }d_{i}{\left(1-\beta \right)}^{i}=0,$$

(51) $$\sum_{i=0}^{\infty }d_{i}{\left(\alpha -\beta \right)}^{i}=\frac{D}{a}$$

and

(52) $$\sum_{i=1}^{n}id_{i}{\left(\alpha -\beta \right)}^{i-1}=0.$$

From Equations (47) and (48), Equations (42), (45), and (46) give

(53) $$\sum_{i=0}^{n}c_{i}{\left(1-\beta \right)}^{i}-\nu \sum_{i=0}^{n}b_{i}{\left(1-\beta \right)}^{i}=0,$$

(54) $$\sum_{i=0}^{\infty }b_{i}{\left(\alpha -\beta \right)}^{i}=\frac{1}{1-\nu }\frac{u_{B}}{b}$$

and

(55) $$\sum_{i=0}^{n}c_{i}{\left(\alpha -\beta \right)}^{i}-\nu \sum_{i=0}^{n}b_{i}{\left(\alpha -\beta \right)}^{i}=\frac{u_{B}}{b}.$$

Eliminating the *u_B_*/*b* from Equations (54) and (55), we obtain

(56) $$\sum_{i=0}^{n}c_{i}{\left(\alpha -\beta \right)}^{i}-\sum_{i=0}^{n}b_{i}{\left(\alpha -\beta \right)}^{i}=0.$$

When *a*, *h*, *E*, *υ*, *D,* and *H* are given in advance, the undetermined constants *b*_0_, *c*_0_, *d*_0_, *d*_1_, and *β* can be determined by simultaneously solving Equations (50)–(53) and (56). Thus, the plate/membrane contact problem under consideration is solved analytically. And, with the known *b*_0_, *c*_0_, *d*_0_, *d*_1_, and *β*, the plate/membrane contact radius *b* (*β* = (1 + *α*)/2 and *α* = *b*/*a*) and all the expressions of stress and deflection can be determined. After considering Equation (37) and *β* = (1 + *α*)/2 = (*a*+ b)/2*a*, from Equations (47)–(49), the dimensional radial stress *σ_r_*, circumferential stress *σ_t_*, and transverse displacement (deflection) *w* may finally be written as

(57) $$\sigma_{r}=E\sum_{i=0}^{\infty }b_{i}{\left(\frac{r}{a}-\frac{a+b}{2a}\right)}^{i},$$

(58) $$\sigma_{t}=E\sum_{i=0}^{\infty }c_{i}{\left(\frac{r}{a}-\frac{a+b}{2a}\right)}^{i}$$

and

(59) $$w=a\sum_{i=0}^{\infty }d_{i}{\left(\frac{r}{a}-\frac{a+b}{2a}\right)}^{i}.$$

### 2.2. Derivation of Input–Output Analytical Relationship of the Rainfall Sensor

As can be seen from Figure 2, the variable capacitor of the circular touch mode capacitive rainfall sensor operates in touch mode, so it can be split into two capacitors in parallel. One of the two is the parallel-plate capacitor in the plate/membrane contact region (0 ≤ *r* ≤ *b*), and the other is composed of two capacitors in series in the plate/membrane non-contact region (*b* ≤ *r* ≤ *a*) (one is the parallel-plate capacitor between the fixed electrode plate and the insulator layer and the other is the non-parallel-plate capacitor between the insulator layer and the deflected movable electrode plate—see Figure 2). Let us denote the relative permittivity of the insulator layer by *ε_r_*_1_, the relative permittivity of the air by *ε_r_*_2_ (about 1.00053), and the vacuum permittivity by *ε*_0_ (about 8.854 × 10^−3^ pF/mm).

The capacitance *C*_1_ of the parallel-plate capacitor in the plate/membrane contact region of 0 ≤ *r* ≤ *b* is given by

(60) $$C_{1}=\frac{\varepsilon_{0}\varepsilon_{r1}\pi b^{2}}{t}.$$

The capacitance *C*_2_ of the non-parallel-plate capacitor in the plate/membrane non-contact region of *b* ≤ *r* ≤ *a* can be derived as follows. If a radial coordinate increment Δ*r* and a circumferential coordinate increment Δ*φ* are taken in the polar plane (*r*, *φ*) to form a micro-area element A′B′C′D′, whose projection on the deflected circular membrane (i.e., the deflected circular conductive membrane (movable electrode plate)) is ABCD, as shown in Figure 3, then, when Δ*r* → 0 and Δ*φ* → 0, the non-parallel-plate capacitor between the insulator layer and the small conductive membrane covered by the projection ABCD may approximately be regarded as a parallel-plate capacitor, and its capacitance may be approximated by

(61) $$\Delta C=\varepsilon_{0}\varepsilon_{r2}\frac{S_{ABCD}}{D-w(r)}=\varepsilon_{0}\varepsilon_{r2}\frac{\frac{\Delta \varphi }{2}{\left(r+\Delta r\right)}^{2}-\frac{\Delta \varphi }{2}r^{2}}{D-w(r)}\approx \varepsilon_{0}\varepsilon_{r2}\frac{r\Delta r\Delta \varphi }{D-w(r)}.$$

Therefore, the capacitance *C*_2_ of the non-parallel-plate capacitor in the plate/membrane non-contact region of *b* ≤ *r* ≤ *a* can calculated by the integration of Equation (54) over the area of *b* ≤ *r* ≤ *a* and 0 ≤ *φ* ≤ 2π

(62) $$C_{2}=\int_{b}^{a}\int_{0}^{2\pi }\varepsilon_{0}\varepsilon_{r2}\frac{r}{D-w(r)}d\varphi dr=2\pi \varepsilon_{0}\varepsilon_{r2}\int_{b}^{a}\frac{r}{D-w(r)}dr.$$

In addition, the capacitance *C*_3_ of the parallel-plate capacitor in the plate/membrane non-contact region of *b* ≤ *r* ≤ *a* is given by

(63) $$C_{3}=\frac{\varepsilon_{0}\varepsilon_{r1}\pi (a^{2}-b^{2})}{t}.$$

Therefore, since the total capacitance *C* of the variable capacitor of the circular touch mode capacitive rainfall sensor in Figure 2 is given by *C* = *C*_1_ + *C*_2-3_ = *C*_1_ + *C*_2_*C*_3_/(*C*_2_ + *C*_3_), where *C*_2-3_ here represents the capacitance after *C*_2_ and *C*_3_ are connected in series, then, from Equations (59), (60), (62), and (63), it may finally be written as

(64) $$C=\frac{\varepsilon_{0}\varepsilon_{r1}\pi b^{2}}{t}+\frac{\frac{\varepsilon_{0}\varepsilon_{r1}\pi {\left(a-b\right)}^{2}}{t}2\pi \varepsilon_{0}\varepsilon_{r2}\int_{b}^{a}\left[r/(D-a\sum_{i=0}^{\infty }d_{i}{\left(\frac{r}{a}-\frac{a+b}{2a}\right)}^{i})\right]dr}{\frac{\varepsilon_{0}\varepsilon_{r1}\pi {\left(a-b\right)}^{2}}{t}+2\pi \varepsilon_{0}\varepsilon_{r2}\int_{b}^{a}\left[r/(D-a\sum_{i=0}^{\infty }d_{i}{\left(\frac{r}{a}-\frac{a+b}{2a}\right)}^{i})\right]dr}.$$

It can be seen from Equation (64) that, as long as the plate/membrane contact radius *b* and the power series coefficients *d_i_* (*i* = 0, 1, 2, 3, *…*) are available, the total capacitance *C* can be determined.

On the other hand, the rainfall in a particular area in a particular amount of time is determined by the total volume of the rainwater that is collected in the storage tank over a given period of time divided by the top opening area of the rainwater collector funnel—see Figure 1. The top opening area of the rainwater collector funnel usually varies according to the particular area, light rain, heavy rain, rainstorm, and other factors, but it is treated as a given parameter. Therefore, the key task that remains here is to determine the total volume *V* of the rainwater that is collected in the storage tank. It can be seen from Figure 2 that the total volume *V* can be calculated by

(65) $$V=\pi a^{2}H+\pi b^{2}D+2\pi \int_{b}^{a}w(r)rdr=\pi a^{2}H+\pi b^{2}D+2\pi \int_{b}^{a}ar\sum_{i=0}^{\infty }d_{i}{\left(\frac{r}{a}-\frac{a+b}{2a}\right)}^{i})dr.$$

It can be seen from Equation (65) that, as long as the plate/membrane contact radius *b* and the power series coefficients *d_i_* (*i* = 0, 1, 2, 3, *…*) are available, the total rainwater volume *V* can be determined.

Since, for the given *a*, *h*, *E*, *υ*, *D,* and *H*, the plate/membrane contact radius *b* and the power series coefficients *d_i_* (*i* = 0, 1, 2, 3, *…*) can be determined by using the analytical solution presented in Section 2.1, the total capacitance *C* and its corresponding total rainwater volume *V* can be determined by Equations (64) and (65). The input–output relationship between the total capacitance *C* and the total rainwater volume *V* of the circular touch mode capacitive rainfall sensor addressed here can thus be determined. Obviously, Equations (64) and (65) are two parametric equations, where the parameter *H*, the height from the polar plane (*r*, *φ*) to the rainwater upper surface (see Figure 2), is a parameter variable associated with the capacitance *C* and the volume *V*. However, between *C* and *V*, which is used as the independent variable and which is used as the dependent variable is not the same, that is, whether *C* is used as the input and *V* as the output, or *V* as the input and *C* as the output. Generally speaking, when describing the input–output relationship of the hardware system of a measurement system, the total rainwater volume *V* is the input and the total capacitance *C* is the output (usually abbreviated as *V–C* relationship), while, when describing the input–output relationship of the whole measurement system, the total capacitance *C* is the input and the total rainwater volume *V* is the output (abbreviated as *C*–*V* relationship).

However, neither the *V*–*C* analytical relationship nor the *C*–*V* analytical relationship can be derived directly from Equations (64) and (65), because it is impossible to eliminate the parameter *H* directly from Equations (64) and (65) (which is included in the dimensionless variable *H*_0_ in the expressions for the power series coefficients *d_i_*—see Equation (37) and Appendix A). The input–output analytical relationship between the total capacitance *C* and the total rainwater volume *V* of the circular touch mode capacitive rainfall sensor addressed here, the *V*–*C* or *C*–*V* analytical relationship, can be given in the form of an explicit expression only by the least-squares data fitting based on a large number of numerical calculations for the total capacitance *C* and its corresponding total rainwater volume *V*, which will be shown in the following section.

## 3. Results and Discussion

In this section, we will focus on some important issues, which are related mainly to the numerical design and calibration of the rainfall sensor and the effect of changing design parameters on the relationship between the input capacitance *C* and rainwater volume *V* of the rainfall sensor.

### 3.1. Numerical Design and Calibration of the Rainfall Sensor

The relevant design parameters of a circular touch mode capacitive rainfall sensor mainly include the following two categories: One of the two is the parameters that need to be specified in advance, including the top opening area of the rainwater collector funnel, the capacity of the cylindrical rainwater storage tank, and so on, which do not need to be considered here. And the other of the two is the parameters that need to be determined by numerical design and calibration, including the radius *a*, thickness *h*, Poisson’s ratio *v* and Young’s modulus of elasticity *E* of the circular conductive membrane (the movable electrode plate), the thickness *t* and relative permittivity *ε*_r1_ of the insulation layer coated on the fixed electrode plate, and the initial parallel gap *D* between the initially flat movable electrode plate and the insulator layer. How to use the analytical solution obtained in Section 2.1 to carry out the numerical design and calibration of a circular touch mode capacitive rainfall sensor is detailed as follows:

The numerical design and calibration of the rainfall sensor begins with a preliminary designation of materials and dimensions.

The so-called preliminary designation of the materials mainly refers to the preliminary selection of the circular conductive membrane (the movable electrode plate, obviously, due to contact with the rainwater, should use thin metal (or conductive polymers) films with antirust properties) and the insulator layer coated on the fixed electrode plate, to preliminarily determine the yield strength *σ*_y_, Poisson’s ratio *v* and Young’s modulus of elasticity *E* of the circular conductive membrane, and the relative permittivity *ε*_r1_ of the insulation layer. Since there may be many materials to choose from, only one of them can be empirically chosen first for the subsequent numerical design.

The so-called preliminary designation of the dimensions mainly refers to the preliminary determination of the values of the inner radius *a* of the cylindrical rainwater storage tank, the thickness *h* of the circular conductive membrane, the thickness *t* of the insulation layer, and the initial parallel gap *D* between the initially flat movable electrode plate and the insulator layer, in which the radius of the initially flat circular conductive membrane (the movable electrode plate) is also equal to the inner radius *a* of the cylindrical rainwater storage tank—see Figure 2.

After the preliminary designation of materials and dimensions, the following numerical calculations can be started. The rain height *H* can start taking its value from a moderately small value, and, then, the values of the undetermined constants *b*_0_, *c*_0_, *d*_0_, *d*_1_, and *β* and the power series coefficients *b_i_* and *c_i_* (*i* = 1, 2, 3, …) and *d_i_* (*i* = 2, 3, 4, *…*) can be calculated by using the analytical solution obtained in Section 2.1. After that, the value of the plate/membrane contact radius *b* can be calculated from *β* = (1 + *α*)/2 = (*a*+ b)/2*a*, the value of the total capacitance *C* can be calculated from Equation (64), and the value of the total rainwater volume *V* can be calculated from Equation (65). Finally, the value of the maximum membrane stress *σ*_m_ can be determined from Equation (57) or (58) (usually, the maximum stress *σ*_m_ occurs in the radial stress; i.e., *σ*_m_ is determined usually by Equation (57)). If the maximum stress *σ*_m_ in this calculation is much less than 0.7 times the yield strength *σ*_y_, then a slightly increased value (small increment) of *H* is needed to repeat the above numerical calculations until the maximum stress *σ*_m_ is close to 0.7*σ*_y_.

Assuming that when the maximum stress *σ*_m_ is close to 0.7*σ*_y_, the maximum value of the rain height *H* is denoted by *H*_m_. If the calculated value of the plate/membrane contact radius *b* (when *H* = *H*_m_) is less than 0.9 times the preliminarily specified value of the radius *a*, and π*a*^2^*H*_m_ is close to and greater than the desired capacity of the cylindrical rainwater storage tank, then the preliminary designation of the circular conductive membrane and its radius *a* and thickness *h* is appropriate and can temporarily meet the design requirements. If π*a*^2^*H*_m_ is much greater than the desired capacity of the cylindrical rainwater storage tank, then the value of the radius *a* needs to be gradually increased (or, alternatively, the value of the thickness *h* is gradually reduced, but there is no need to change the material of the conductive membrane) and the above numerical calculation is repeated until π*a*^2^*H*_m_ is close to and greater than the desired capacity of the cylindrical rainwater storage tank and the plate/membrane contact radius *b* (when *H* = *H*_m_) is less than 0.9 times the radius *a*. Obviously, if π*a*^2^*H*_m_ is much less than the desired capacity of the cylindrical rainwater storage tank, then the value of the radius *a* needs to be gradually reduced (or, alternatively, the value of the thickness *h* is gradually increased) and the above numerical calculation is repeated until π*a*^2^*H*_m_ is close to and greater than the capacity specified in advance of the cylindrical rainwater storage tank, and the plate/membrane contact radius *b* (when *H* = *H*_m_) is less than 0.9 times the radius *a*.

After that, the numerical calibration of the input–output analytical relationship of the circular touch mode capacitive rainfall sensor can be started. Obviously, since the input–output relationship of the whole rainfall measurement system needs to be described, the total capacitance *C* should be the input variable while the total rainwater volume *V* should be the output variable; the input–output analytical relationship can be abbreviated as the *C*–*V* analytical relationship. As stated in Section 2.2, the *C*–*V* analytical relationship can be given in the form of an explicit expression only by the least-squares data fitting based on a large number of numerical calculations for the total capacitance *C* and its corresponding total rainwater volume *V*. Therefore, the slightly increased value of *H* in the above numerical calculations should be an increment as small as possible to produce as many numerical calculation values as possible, thus ensuring the fitting accuracy of the fitted *C*–*V* analytical relationship.

If the *C*–*V* analytical relationship fitted does not meet the requirements (for example, the variation range of the input capacitance *C* is too narrow, or the value of rainwater volume *V* corresponding to one unit of capacitance is too large), then some design parameters need to be adjusted to meet the requirements. First, consider the adjustment of the thickness *t* of the insulation layer or the initial parallel gap *D* between the initially flat movable electrode plate and the insulator layer: if the requirements are still not satisfied, then the radius *a* or thickness *h* of the circular conductive membrane needs to be further adjusted, and even the Poisson’s ratio *v* and Young’s modulus of elasticity *E* (changing the material of the conductive membrane). Then, exactly which one or some of the design parameters need to be adjusted, and how to adjust these parameters (whether to make them bigger or smaller) are questions that depend on the specific situation of the fitted *C*–*V* analytical relationship, especially how changing the design parameters affects the fitted *C*–*V* analytical relationship (see the following section for details).

In the following, an example is given to illustrate how to carry out the numerical design and calibration of the rainfall sensor. Suppose that the required or desired capacity of the cylindrical rainwater storage tank is about 15,000,000 mm^3^, and a circular conductive membrane with radius *a* = 70 mm, thickness *h* = 0.3 mm, Poisson’s ratio *v* = 0.45, and Young’s modulus of elasticity *E* = 3.05 MPa is used as the movable electrode plate of the variable capacitor of the circular touch mode capacitive rainfall sensor—see Figure 2. The insulator layer is assumed to be 0.1 mm-thick polystyrene; then, *t* = 0.1 mm and *ε*_r1_ = 2.7, and the initial parallel gap between the initially flat movable electrode plate and the insulator layer is preliminarily specified as *D* = 10 mm. In addition, the air relative permittivity is *ε*_r2_ = 1.00053, and the vacuum permittivity is *ε*_0_ = 8.854 × 10^−3^ pF/mm. After the above preliminary designation of materials and dimensions, the numerical calculations are started from the rainwater height *H* = 8 mm, and stopped when *H* = 1000 mm. The numerical calculation values of the undetermined constants *β, b*_0_, *c*_0_, *d*_0_, and *d*_1_, the maximum membrane stress *σ*_m_, the capacitance *C*_1_ of the plate/membrane contact region of 0 ≤ *r* ≤ *b*, the capacitance *C*_2-3_ of the plate/membrane non-contact region of *b* ≤ *r* ≤ *a* (where *C*_2-3_ = *C*_2_*C*_3_/(*C*_2_ + *C*_3_), the capacitance after *C*_2_ and *C*_3_ are connected in series—see Equations (62) and (63)), the total capacitance *C* of the rainfall sensor, the volume *V,* and height *H* of the collected rainwater in the storage tank (see Figure 2) are listed in Table 1.

**Figure 6:**
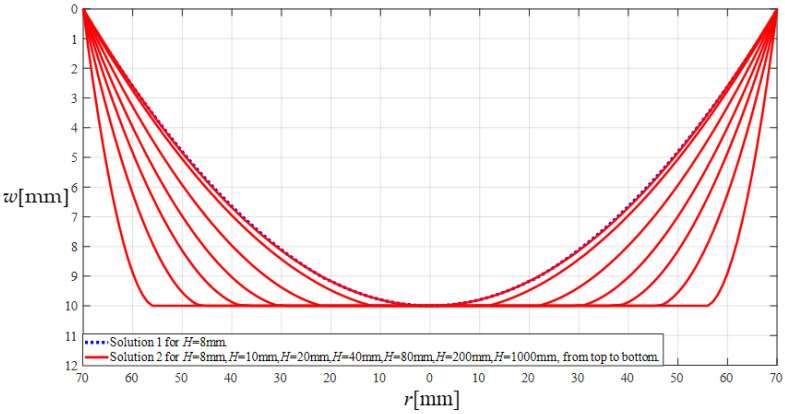
The variation in the deflection *w* with the coordinate variable *r*, where the dashed line (“Solution 1”) refers to the calculated result by the analytical solution for the plate/membrane non-contact problem in Section 2 in [51], where the rainwater height *H* takes only 8 mm; and the solid line (“Solution 2”) refers to the calculated results by the analytical solution for the plate/membrane contact problem in Section 2 in this paper, where the rainwater height *H* takes 8 mm, 10 mm, 20 mm, 40 mm, 80 mm, 200 mm, and 1000 mm, respectively.

The effectiveness of the above calculations can be validated as follows: The plate/membrane contact radius *b* is given by *b* = *a*(2*β*−1), Then, the *β* values in Table 1 can be used to determine the corresponding values of the plate/membrane contact radius *b*. The power series coefficients *b_i_* and *c_i_* (*i* = 1, 2, 3, …) and *d_i_* (*i* = 2, 3, 4, *…*) are expressed as polynomials with regard to the undetermined constants *b*_0_, *c*_0_, *d*_0_, *d*_1_, and *β* and the dimensionless variables *H*_0_ = *H*/*a* and *G* = *ρga*^2^/*E*/*h*, as shown in Equation (37) and Appendix A. Therefore, the values of *b*_0_, *c*_0_, *d*_0_, *d*_1_, and *β* in Table 1 can be used to determine the corresponding values of the power series coefficients *b_i_* and *c_i_* (*i* = 1, 2, 3, …) and *d_i_* (*i* = 2, 3, 4, *…*), and the corresponding deflection expressions *w*(*r*) (in which *b* ≤ *r* ≤ *a*) can be determined by Equation (59). Figure 6 shows the shapes of the determined deflection curve *w*(*r*) corresponding to *H* = 8 mm, *H* = 10 mm, *H* = 20 mm, *H* = 40 mm, *H* = 80 mm, *H* = 200 mm, and *H* = 1000 mm, respectively, where the seven solid lines (“Solution 2”) are the results calculated by using the analytical solution for the plate/membrane contact problem in Section 2.1 in this paper, while the dashed line (“Solution 1”) is the result calculated by using the analytical solution for the plate/membrane non-contact problem in Section 2 in [51].

The deflection expression *w*(*r*) of the dashed line (“Solution 1”) in Figure 6 is determined as follows: The power series coefficients *c_i_* and *d_i_* (*i* = 1, 2, 3, …) are expressed as polynomials with regard to the undetermined constants *c*_0_ and *d*_0_ and the dimensionless variables *H*_0_ = *H*/*a* and *G* = *ρga*^2^/*E*/*h*, as shown in Equation (37) and Appendices A and B in [51]. When *H* = 8 mm, *a* = 70 mm, *h* = 0.3 mm, *E* = 3.05 MPa, *v* = 0.45, *ρ* = 10^−6^ kg/mm^3^, and *g* = 9.8 N/kg, the values of the dimensionless variables *H*_0_ and *G* are about *H*_0_ = 0.1142857143 and *G* = 0.0524808743, respectively, and the values of the undetermined constants *c*_0_ and *d*_0_ can be determined by simultaneously solving Equations (26) and (27) in [51] and are about *c*_0_ = 0.0234613886 and *d*_0_ = 0.1428600005. Therefore, from Equations (17) and (25) in [51], the deflection expression *w*(*r*) of the dashed line (“Solution 1”) in Figure 6 can be finally written as

(66) $$\begin{array}{ll} w(r) & =10.00020004-0.002076449712r^{2}+0.000000003801071338r^{4}\\ & +5.618369296\times 10^{-13}r^{6}+2.486496669\times 10^{-17}r^{8}+8.501687411\times 10^{-22}r^{10}\\ & +2.119790950\times 10^{-26}r^{12}+1.651234643\times 10^{-31}r^{14}-2.077126600\times 10^{-35}r^{16}\\ & -1.607089683\times 10^{-39}r^{18}-7.140884646\times 10^{-44}r^{20} \end{array},$$

where 0 ≤ *r* ≤ 70 mm.

It can be seen from Figure 6 that, in the plate/membrane contact problem (the seven solid lines (“Solution 2”) in Figure 6), when the rainwater height is reduced from *H* = 1000 mm to *H* = 8 mm, the shape of the deflection curve shows a relatively regular change, where the plate/membrane contact radius *b* when *H* = 8 mm is *b* = *a*(2*β*−1) = 70 mm × (2 × 0.5010245 − 1) = 0.14343 mm. And, in the plate/membrane non-contact problem (the dashed line (“Solution 1”) in Figure 6), the maximum deflection *w_m_* (*w* at *r* = 0 mm) when *H* = 8 mm is about *w_m_* = 10.00020004 mm—see Equation (66). The dashed line for *H* = 8 mm in Figure 6 seems to coincide very well with the solid line for *H* = 8 mm in Figure 6, while the same circular membrane elastically behaves continuously before and after it is in contact with the insulator layer. Therefore, the analytical solution describing the non-contact elastic behavior, i.e., the analytical solution for the plate/membrane non-contact problem in [51], and the analytical solution describing the contact elastic behavior, i.e., the analytical solution for the plate/membrane contact problem in this paper, are mathematically coordinated and continuous. This suggests that, if the analytical solution describing the non-contact elastic behavior in [51] is reliable (correct), then the analytical solution describing the contact elastic behavior in this paper is also reliable (correct); otherwise, there is no coordination between the two. Since the analytical solution describing the non-contact elastic behavior has been proven to be reliable (correct) by the confirmatory experiment in Section 3.4 in [51] (see Figure 12 and Table 3 in [51]), then the analytical solution for the plate/membrane contact problem in Section 2.1 in this paper is reliable (correct) to a certain extent, and, thus, the above calculations are valid and reliable.

**Figure 7:**
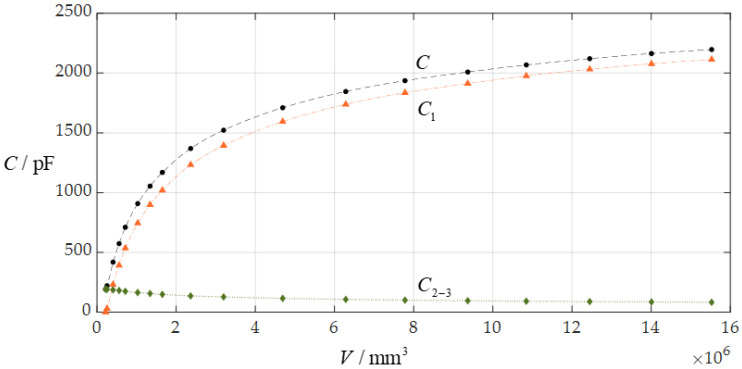
The relationship between the capacitance *C*_1_ of the plate/membrane contact region (0 ≤ *r* ≤ *b*), the capacitance *C*_2-3_ of the plate/membrane non-contact region (*b* ≤ *r* ≤ *a* and *C*_2-3_ = *C*_2_*C*_3_/(*C*_2_ + *C*_3_)), and the total capacitance *C* when *a* = 70 mm, *h* = 0.3 mm, *t* = 0.1 mm, *E* = 3.05 MPa, *ν* = 0.45, and *g* = 10 mm.

Now, let us analyze the input–output relationship of the rainwater volume *V* as the input and total capacitance *C* as the output, i.e., the *V*-*C* relationship, to understand the characteristics of the hardware system of this circular touch mode capacitive rainfall sensor. To this end, we plot the numerical values of the capacitance *C*_1_, *C*_2-3_ (*C*_2-3_ = *C*_2_*C*_3_/(*C*_2_ + *C*_3_)), and *C*, and rainwater volume *V* in Table 1 as a scatter plot, to investigate the respective contributions of the capacitance *C*_1_ in the plate/membrane contact region (0 ≤ *r* ≤ *b*) and capacitance *C*_2-3_ in the plate/membrane non-contact region (*b* ≤ *r* ≤ *a*) to the total capacitance *C*, as shown in Figure 7. It can be seen from Figure 7 that the capacitance *C*_1_ of the plate/membrane contact region (0 ≤ *r* ≤ *b*) has achieved a dominant position in the total capacitance *C*, while the share of the capacitance *C*_2-3_ of the plate/membrane non-contact region (*b* ≤ *r* ≤ *a*) in the total capacitance *C* is very small. This is what makes this circular touch mode capacitive rainfall sensor special.

On the other hand, a comparison between non-touch mode operation and touch mode operation can show that the circular capacitive rainfall sensor operating in contact mode can provide a better application performance than the same circular capacitive rainfall sensor operating in non-contact mode. It can be seen from Table 1 and Figure 7 that, in comparison with non-touch mode operation, the ranges of both the input capacitance *C* and the output rainwater volume *V* are greatly increased, thus providing a larger application space. When the circular capacitive rainfall sensor operates in touch mode, its input range of capacitance *C* is 2196.805 pF − 192.233 pF = 2004.572 pF, its output range of rainwater volume *V* is 15,526,942.340 mm^3^ − 215,248.882 mm^3^ = 15,311,693.458 mm^3^, and its average increase in the maximum membrane stress *σ*_m_ per unit rainwater volume *V* is equal to (0.344 MPa − 0.075 MPa)/15,311,693.458 mm^3^ ≈ 1.757 × 10^−8^ MPa/mm^3^. When the same circular capacitive rainfall sensor operates in non-touch mode, its maximum input of capacitance *C* is about 192.233 pF, its maximum output of rainwater volume *V* is about 215,248.882 mm^3^, and its average increase in the maximum membrane stress *σ*_m_ per unit rainwater volume *V* is as high as 0.075 MPa/215,248.882 mm^3^ ≈ 34.843 × 10^−8^ MPa/mm^3^ (which is about 20 times 1.757 × 10^−8^ MPa/mm^3^).

Obviously, when the circular capacitive rainfall sensor operates in contact mode, the rainwater in the rainwater storage tank is borne by both the moveable electrode plate (circular conductive membrane) and the fixed electrode plate, and the more rainwater in the rainwater storage tank, the more rainwater borne by the fixed electrode plate, and the less rainwater borne by the circular conductive membrane, thus greatly improving the rainwater carrying capacity of the rainfall sensor—see Figure 2. This is why the average increase in the maximum membrane stress *σ*_m_ per unit rainwater volume *V* of the circular capacitive rainfall sensor operating in touch mode is much less than that of the same circular capacitive rainfall sensor operating in non-touch mode. This is where the circular capacitive rainfall sensor operating in touch mode excels.

**Figure 8:**
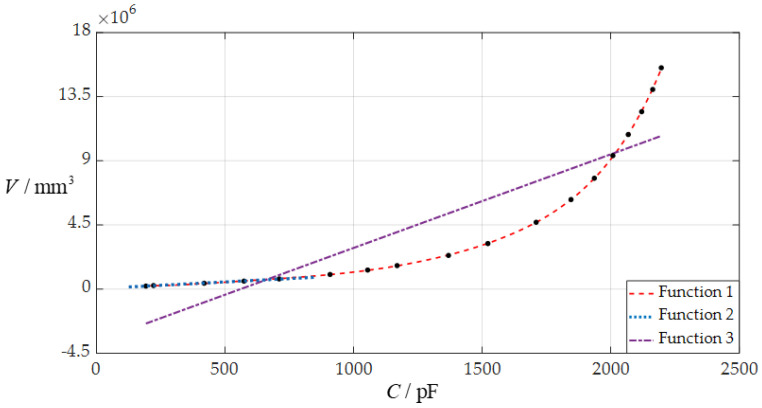
The three *C–V* analytical relationships fitted to the numerical calculation values of the total capacitance *C* and rainwater volume *V* in Table 1 when *a* = 70 mm, *h* = 0.3 mm, *t* = 0.1 mm, *E* = 3.05 MPa, *ν* = 0.45, and *D* = 10 mm, where Function 1 refers to the *C–V* analytical relationship fitted by a curve, Functions 2 and 3 refer to the *C–V* analytical relationships fitted by two straight lines, and the fitted analytical expressions of Functions 1, 2, and 3 are shown in Table 2.

Now, let us use the least-squares method to carry out the least-squares data fitting for the numerical values of the total capacitance *C* and rainwater volume *V* in Table 1, as shown in Figure 8, where the scatter points are generated by the data of the capacitance *C* and volume *V* in Table 1, the capacitance *C* is used as the input, the rainwater volume *V* is used as the output, “Function 1” refers to the *C–V* analytical relationship fitted by a curve, and “Function 2” and “Function 3” refer to the *C–V* analytical relationship fitted by two straight lines. The fitted analytical expressions of Functions 1, 2, and 3 are listed in Table 2.

It can be seen from Figure 8 and Table 2 that, from the point of view of the acceptable levels of the fitted errors in Table 2, the circular touch mode capacitive rainfall sensor to be designed can use “Function 1” or “Function 2” as its *C–V* analytical relationship, and cannot use “Function 3”. As can be seen from Table 2, the linear *C–V* analytical relationship, “Function 2”, is simple and the nonlinear *C–V* analytical relationship, “Function 1”, is complex, showing that “Function 1” applies only to digital techniques while “Function 2” applies to both digital techniques and analog techniques. However, it can also be seen from Figure 8 and Table 2 that the variation ranges of both the input capacitance *C* and the output rainwater volume *V* are too narrow, which may not meet the requirements for application or technique and need to be increased or adjusted.

In fact, the nonlinear *C–V* analytical relationship, “Function 1” in Figure 8 and Table 2, may also not meet the requirements for application or technique and need to be adjusted. Therefore, it is often necessary to adjust (increase or decrease) the variation range of the input capacitance *C* or output rainwater volume *V*, and sometimes even the variation ranges of both the input capacitance *C* and the output rainwater volume *V*. Such an adjustment can be achieved by changing some or just one of the design parameters, such as the radius *a*, thickness *h*, Poisson’s ratio *v* and Young’s modulus of elasticity *E* of the circular membrane, the thickness *t* of the insulation layer, and the initial parallel gap *D* between the initially flat movable electrode plate and the insulator layer. In this case, it is necessary to know in advance how changing each design parameter will affect the variation range of the input capacitance *C* or output rainwater volume *V* (whether to make the variation range bigger or smaller); i.e., it is necessary to know in advance the effect of changing design parameters on the *C*–*V* relationships, which is addressed in the following section.

### 3.2. The Effect of Changing Design Parameters on the C–V Relationship of the Rainfall Sensor

**Figure 9:**
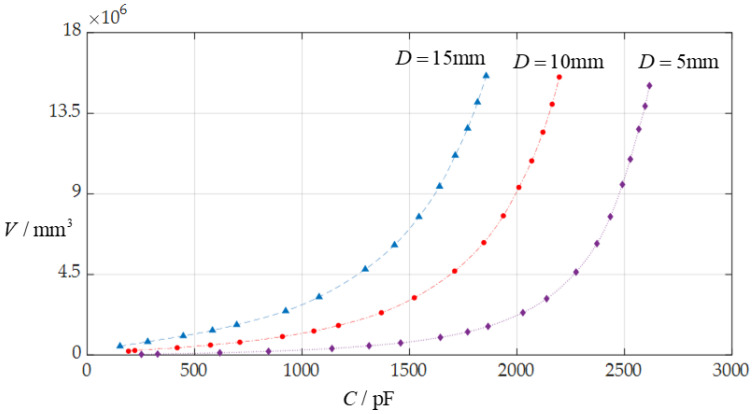
The effect of changing the initially parallel gap *D* on *C*–*V* relationship when *a* = 70 mm, *h* = 0.3 mm, *t* = 0.1 mm, *E* = 3.05 MPa, *ν* = 0.45, and *D* takes 5 mm, 10 mm, and 15 mm, respectively.

**Figure 10:**
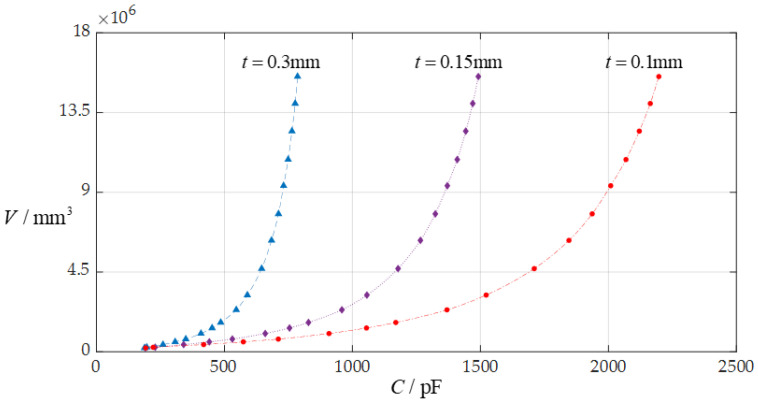
The effect of changing the insulator layer thickness *t* on the *C*–*V* relationship when *a* = 70 mm, *D* = 10 mm, *h* = 0.3 mm, *E* = 3.05 MPa, *ν* = 0.45, and *t* takes 0.1 mm, 0.15 mm, and 0.3 mm, respectively.

**Figure 11:**
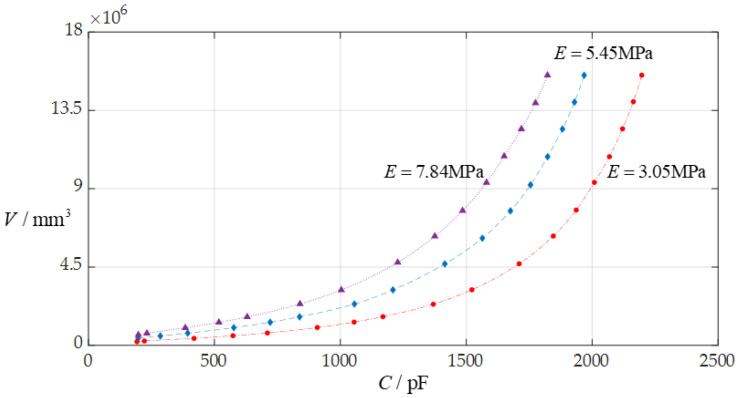
The effect of changing the Young’s modulus of elasticity *E* on the *C*–*V* relationship when *a* = 70 mm, *D* = 10 mm, *h* = 0.3 mm, *t* = 0.1 mm, *ν* = 0.45, and *E* takes 7.84 MPa, 5.45 MPa, and 3.05 MPa, respectively.

**Figure 12:**
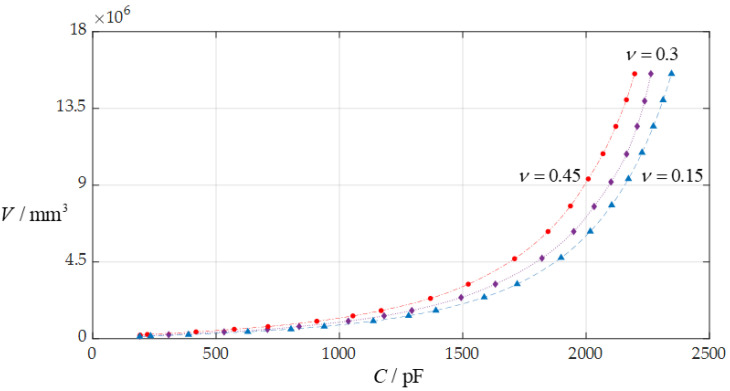
The effect of changing the Poisson’s ratio *v* on the *C*–*V* relationship when *a* = 70 mm, *D* = 10 mm, *h* = 0.3 mm, *t* = 0.1 mm, *E* = 3.05 MPa, and *v* takes 0.45, 0.3, and 0.15, respectively.

**Figure 13:**
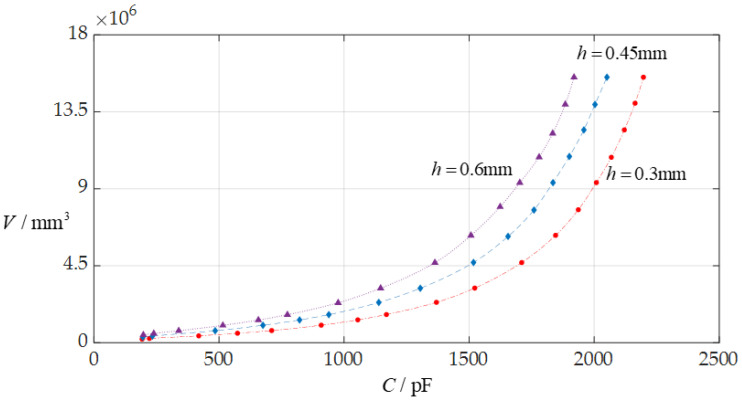
The effect of changing the membrane thickness *h* on the *C*–*V* relationship when *a* = 70 mm, *D* = 10 mm, *t* = 0.1 mm, *E* = 3.05 MPa, *ν* = 0.45, and *h* takes 0.3 mm, 0.45 mm, and 0.6 mm, respectively.

**Figure 14:**
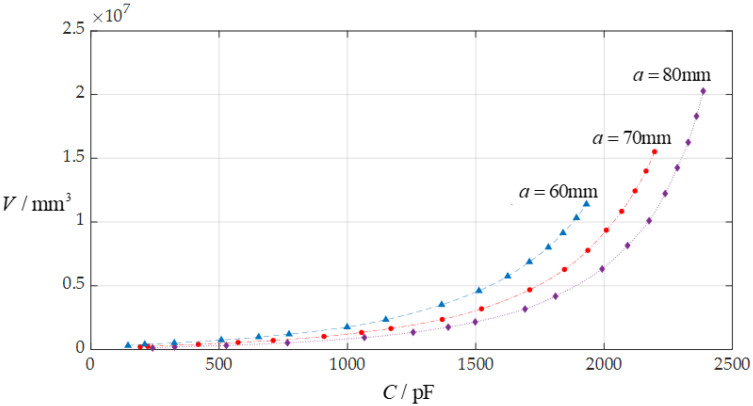
The effect of changing the circular membrane radius *a* on the *C*–*V* relationship when *D* = 10 mm, *h* = 0.3 mm, *t* = 0.1 mm, *E* = 3.05 MPa, *ν* = 0.45, and *a* takes 80 mm, 70 mm, and 60 mm, respectively.

In this section, each design parameter takes three successively increasing or decreasing values for numerical calculation, where one of the three valves taken by each design parameter is the one used in Section 3.1 (i.e., *D* = 10 mm, *a* = 70 mm, *h* = 0.3 mm, *E* = 3.05 MPa, *v* = 0.45, *t* = 0.1 mm, *ε*_r2_ = 1.00053, *ε*_r1_ = 2.5, and *ε*_0_ = 8.854 × 10^−3^ pF/mm, respectively). And, then, the effect of successively increasing or decreasing the values of each design parameter on the ranges of the input capacitance *C* and output rainwater volume *V* is investigated, respectively, as shown in Figure 9, Figure 10, Figure 11, Figure 12, Figure 13 and Figure 14.

From Figure 9, Figure 10, Figure 11, Figure 12, Figure 13 and Figure 14, the following can be concluded: ① the decrease in the initially parallel gap *D* can increase the range of the input capacitance *C* while the range change in the rainwater volume *V* is not obvious; ② the decrease in the insulator layer thickness *t* does not change the range of the output rainwater volume *V* but can increase the range of the input capacitance *C*; ③ the decrease in the Young’s modulus of elasticity *E* can increase the range of the input capacitance *C*, but the range change in the output rainwater volume *V* is not obvious; ④ the decrease in the Poisson’s ratio *v* does not change the range of the output rainwater volume *V* but can increase the range of the input capacitance *C*; ⑤ the decrease in the thickness *h* of the circular membrane can increase the range of both the input capacitance *C* and the output rainwater volume *V*; and ⑥ the increase in the radius *a* of the circular membrane can increase the ranges of both the output rainwater volume *V* and the input capacitance *C*.

## 4. Concluding Remarks

In this paper, a theoretical study on the circular capacitive rainfall sensor operating in touch mode is presented for the first time. From this study, the following conclusions can be drawn:

For the circular touch mode capacitive rainfall sensor, the capacitance *C*_1_ of its plate/membrane contact region occupies a dominant position in its total capacitance *C*, while the share of the capacitance *C*_2-3_ of its plate/membrane non-contact region in its total capacitance *C* is very small.

In comparison with non-touch mode operation, touch mode operation can greatly reduce the average increase in the maximum membrane stress *σ*_m_ per unit rainwater volume *V* of the rainfall sensor, thus greatly improving the rainwater carrying capacity of the rainfall sensor.

The variation range of the input capacitance *C* of the circular touch mode capacitive rainfall sensor can be increased by the decrease in the initially parallel gap *D*, insulator layer thickness *t*, Young’s modulus of elasticity *E*, Poisson’s ratio *v*, or membrane thickness *h*, or by the increase in the radius *a* of the circular conductive membrane (the movable electrode plate).

The variation range of the output rainwater volume *V* of the circular touch mode capacitive rainfall sensor can be increased by the decrease in the thickness *h* of the circular conductive membrane (the movable electrode plate), or by the increase in the radius *a* of the circular membrane.

The present work can be further extended to the experimental study and development of the circular touch mode capacitive rainfall sensor.

## Figures and Tables

**Table 1:** The numerical calculation results of the undetermined constants *β, b*_0_, *c*_0_, *d*_0_, and *d*_1_, maximum stress *σ*_m_, capacitance *C*_1_, capacitance *C*_2-3_, total capacitance *C,* and rainwater volume *V* when *a* = 70 mm, *h* = 0.3 mm, *t* = 0.1 mm, *E* = 3.05 MPa, *v* = 0.45, *D* = 10 mm, *ρ* = 10^−6^ kg/mm^3^, and *g* = 9.8 N/kg, and the rainwater height *H* takes different values.

*V*/mm^3^	*H*/mm	*β*	*b*_0_	*c*_0_	*d*_0_	*d*_1_	*σ*_m_/MPa	*C*_1_/pF	*C*_2-3_/pF	*C*/pF
215,248.882	8	0.5010245	0.0220436	0.0193413	0.1059553	−0.1464721	0.075	0.014	192.219	192.233
253,117.905	10.0	0.5473127	0.0234119	0.0203047	0.1033247	−0.1617807	0.075	30.511	191.207	221.717
414,157.880	20.0	0.6303382	0.0290129	0.0253605	0.1029199	−0.1985574	0.091	231.547	187.294	418.840
561,001.880	30.0	0.6695382	0.0332550	0.0295802	0.1033055	−0.2178493	0.103	391.769	181.981	573.750
715,132.156	40.0	0.6982443	0.0365297	0.0324075	0.1037764	−0.2423645	0.113	535.668	174.829	710.498
1,028,637.040	60.0	0.7336455	0.0419989	0.0375438	0.1042676	−0.2738678	0.130	744.062	164.421	908.484
1,340,303.982	80.0	0.7567581	0.0464284	0.0416989	0.1045804	−0.2993594	0.143	898.552	156.153	1054.705
1,650,985.625	100.0	0.7735285	0.0502065	0.0452393	0.1048003	−0.3211105	0.154	1019.765	149.426	1169.191
2,364,872.625	150.0	0.8007285	0.0580155	0.0524622	0.1051548	−0.3624782	0.179	1232.662	136.431	1369.093
3,198,261.376	200.0	0.8198436	0.0640878	0.0582248	0.1053642	−0.4011219	0.197	1394.344	127.949	1522.294
4,689,985.376	300.0	0.8420126	0.0739524	0.0670015	0.1056242	−0.4582146	0.226	1594.333	115.743	1710.076
6,283,660.204	400.0	0.8571290	0.0817892	0.0747527	0.1057796	−0.5033008	0.250	1738.381	107.279	1845.660
7,779,456.204	500.0	0.8669820	0.0883945	0.0808515	0.1058955	−0.5422184	0.270	1835.626	100.830	1936.456
9,365,758.565	600.0	0.8745939	0.0942722	0.0863970	0.1059707	−0.5753972	0.288	1912.565	96.089	2008.653
10,844,109.565	700.0	0.8807977	0.0993213	0.0913549	0.1060326	−0.6028149	0.303	1976.438	91.965	2068.404
12,446,629.010	800.0	0.8860938	0.1042310	0.0956829	0.1060879	−0.6329380	0.319	2031.797	88.686	2120.483
14,006,297.102	900.0	0.8904238	0.1085215	0.0996215	0.1061205	−0.6598215	0.332	2077.625	85.808	2163.433
15,526,942.340	1000.0	0.8937901	0.1126503	0.1035316	0.1061697	−0.6815884	0.344	2113.607	83.198	2196.805

**Table 2:** The fitted analytical expressions of the Functions 1, 2, and 3 in Figure 8.

Functions	*C*/pF	*V*/mm^3^	Analytical Expressions	Fitted Error (Mean Absolute Error)
Function 1	192.233~2196.805	215,248.882~15,526,942.340	*V =* 2.070607 × 10^−12^*C*^6^ − 1.209120 × 10^−8^*C*^5^ + 2.855011 × 10^−5^*C*^4^ − 3.291677 × 10^−2^*C*^3^ + 1.974305 × 10 *C*^2^ − 4.781130 × 10^3^*C* + 6.208878 × 10^5^	1.83%
Function 2	192.233~710.498	215,248.882~715,132.156	*V* = 938.65*C* + 34,300.05	2.35%
Function 3	192.233~2196.805	215,248.882~15,526,942.340	*V* = 6.564548 × 10^3^*C* − 3.673163 × 10^6^	192.58%

## Data Availability

Data are contained within the article.

## Appendix A

$$b_{1}=-\frac{b_{0}-c_{0}}{\beta }$$

$$b_{2}=-\frac{2b_{1}-c_{1}}{2\beta }$$

$$b_{3}=-\frac{3b_{2}-c_{2}}{3\beta }$$

$$b_{4}=-\frac{4b_{3}-c_{3}}{4\beta }$$

$$b_{5}=-\frac{5b_{4}-c_{4}}{5\beta }$$

$$b_{6}=-\frac{6b_{5}-c_{5}}{6\beta }$$

$$b_{7}=-\frac{7b_{6}-c_{6}}{7\beta }$$

$$b_{8}=-\frac{8b_{7}-c_{7}}{8\beta }$$

$$b_{9}=-\frac{9b_{8}-c_{8}}{9\beta }$$

$$b_{10}=-\frac{10b_{9}-c_{9}}{10\beta }$$

$$b_{11}=-\frac{11b_{10}-c_{10}}{11\beta }$$

$$b_{12}=-\frac{12b_{11}-c_{11}}{12\beta }$$

$$b_{13}=-\frac{13b_{12}-c_{12}}{13\beta }$$

$$b_{14}=-\frac{14b_{13}-c_{13}}{14\beta }$$

$$b_{15}=-\frac{15b_{14}-c_{14}}{15\beta }$$

$$b_{16}=-\frac{16b_{15}-c_{15}}{16\beta }$$

$$c_{1}=\frac{2v\beta b_{1}+2vb_{0}-2vc_{0}-{d_{1}}^{2}+2b_{0}-2c_{0}}{2\beta }$$

$$c_{2}=\frac{2v\beta b_{2}+2vb_{1}-vc_{1}-2d_{1}d_{2}+b_{1}-2c_{1}}{2\beta }$$

$$c_{3}=\frac{3v\beta b_{3}+3vb_{2}-vc_{2}-3d_{1}d_{3}-2{d_{2}}^{2}+b_{2}-3c_{2}}{3\beta }$$

$$c_{4}=\frac{4\beta vb_{4}+4vb_{3}-vc_{3}-4d_{1}d_{4}-6d_{2}d_{3}+b_{3}-4c_{3}}{4\beta }$$

$$c_{5}=\frac{10\beta vb_{5}+10vb_{4}-2vc_{4}-10d_{1}d_{5}-16d_{2}d_{4}-9{d_{3}}^{2}+2b_{4}-10c_{4}}{10\beta }$$

$$c_{6}=\frac{6\beta vb_{6}+6vb_{5}-vc_{5}-6d_{1}d_{6}-10d_{2}d_{5}-12d_{3}d_{4}+b_{5}-6c_{5}}{6\beta }$$

$$c_{7}=\frac{7\beta vb_{7}+7vb_{6}-vc_{6}-7d_{1}d_{7}-12d_{2}d_{6}-15d_{3}d_{5}-8{d_{4}}^{2}+b_{6}-7c_{6}}{7\beta }$$

$$c_{8}=\frac{8\beta vb_{8}+8vb_{7}-vc_{7}-8d_{1}d_{8}-14d_{2}d_{7}-18d_{3}d_{6}-20d_{4}d_{5}+b_{7}-8c_{7}}{8\beta }$$

$$c_{9}=\frac{18\beta vb_{9}+18vb_{8}-2vc_{8}-18d_{1}d_{9}-32d_{2}d_{8}-42d_{3}d_{7}-48d_{4}d_{6}-25{d_{5}}^{2}+2b_{8}-18c_{8}}{18\beta }$$

$$c_{10}=\frac{10\beta vb_{10}+10vb_{9}-vc_{9}-10d_{1}d_{10}-18d_{2}d_{9}-24d_{3}d_{8}-28d_{4}d_{7}-30d_{5}d_{6}+b_{9}-10c_{9}}{10\beta }$$

$$\begin{array}{ll} c_{11} & =\frac{1}{11\beta }(11\beta vb_{11}+11vb_{10}-vc_{10}-11d_{1}d_{11}-20d_{2}d_{10}-27d_{3}d_{9}-32d_{4}d_{8}\\ & -35d_{5}d_{7}-18{d_{6}}^{2}+b_{10}-11c_{10}) \end{array}$$

$$\begin{array}{ll} c_{12} & =\frac{1}{12\beta }(12\beta vb_{12}+12vb_{11}-vc_{11}-12d_{1}d_{12}-22d_{2}d_{11}-30d_{3}d_{10}-36d_{4}d_{9}\\ & -40d_{5}d_{8}-42d_{6}d_{7}+b_{11}-12c_{11}) \end{array}$$

$$\begin{array}{ll} c_{13} & =\frac{1}{26\beta }(26\beta vb_{13}+26vb_{12}-2vc_{12}-26d_{1}d_{13}-48d_{2}d_{12}-66d_{3}d_{11}-80d_{4}d_{10}\\ & -90d_{5}d_{9}-96d_{6}d_{8}-49{d_{7}}^{2}+2b_{12}-26c_{12}) \end{array}$$

$$\begin{array}{ll} c_{14} & =\frac{1}{14\beta }(14\beta vb_{14}+14vb_{13}-vc_{13}-14d_{1}d_{14}-26d_{2}d_{13}-36d_{3}d_{12}-44d_{4}d_{11}\\ & -50d_{5}d_{10}-54d_{6}d_{9}-56d_{7}d_{8}+b_{13}-14c_{13}) \end{array}$$

$$\begin{array}{ll} c_{15} & =\frac{1}{15\beta }(15\beta vb_{15}+15vb_{14}-vc_{14}-15d_{1}d_{15}-28d_{2}d_{14}-39d_{3}d_{13}-48d_{4}d_{12}\\ & -55d_{5}d_{11}-60d_{6}d_{10}-63d_{7}d_{9}-32{d_{8}}^{2}+b_{14}-15c_{14}) \end{array}$$

$$\begin{array}{ll} c_{16} & =\frac{1}{16\beta }(16\beta vb_{16}+16vb_{15}-vc_{15}-16d_{1}d_{16}-30d_{2}d_{15}-42d_{3}d_{14}-52d_{4}d_{13}\\ & -60d_{5}d_{12}-66d_{6}d_{11}-70d_{7}d_{10}-72d_{8}d_{9}+b_{15}-16c_{15}) \end{array}$$

$$d_{2}=-\frac{G\beta H_{0}+G\beta d_{0}+\beta b_{1}d_{1}+b_{0}d_{1}}{2\beta b_{0}}$$

$$d_{3}=-\frac{G\beta d_{1}+4\beta b_{1}d_{2}+2\beta b_{2}d_{1}+GH_{0}+Gd_{0}+4b_{0}d_{2}+2b_{1}d_{1}}{6\beta b_{0}}$$

$$d_{4}=-\frac{G\beta d_{2}+9\beta b_{1}d_{3}+6\beta b_{2}d_{2}+3\beta b_{3}d_{1}+Gd_{1}+9b_{0}d_{3}+6b_{1}d_{2}+3b_{2}d_{1}}{12\beta b_{0}}$$

$$\begin{array}{ll} d_{5} & =-\frac{1}{20\beta b_{0}}(G\beta d_{3}+16\beta b_{1}d_{4}+12\beta b_{2}d_{3}+8\beta b_{3}d_{2}+4\beta b_{4}d_{1}+Gd_{2}\\ & +16b_{0}d_{4}+12b_{1}d_{3}+8b_{2}d_{2}+4b_{3}d_{1}) \end{array}$$

$$\begin{array}{ll} d_{6} & =-\frac{1}{30\beta b_{0}}(G\beta d_{4}+25\beta b_{1}d_{5}+20\beta b_{2}d_{4}+15\beta b_{3}d_{3}+10\beta b_{4}d_{2}+5\beta b_{5}d_{1}\\ & +Gd_{3}+25b_{0}d_{5}+20b_{1}d_{4}+15b_{2}d_{3}+10b_{3}d_{2}+5b_{4}d_{1}) \end{array}$$

$$\begin{array}{ll} d_{7} & =-\frac{1}{42\beta b_{0}}(G\beta d_{5}+36\beta b_{1}d_{6}+30\beta b_{2}d_{5}+24\beta b_{3}d_{4}+18\beta b_{4}d_{3}+12\beta b_{5}d_{2}\\ & +6\beta b_{6}d_{1}+Gd_{4}+36b_{0}d_{6}+30b_{1}d_{5}+24b_{2}d_{4}+18b_{3}d_{3}+12b_{4}d_{2}+6b_{5}d_{1}) \end{array}$$

$$\begin{array}{ll} d_{8} & =-\frac{1}{56\beta b_{0}}(G\beta d_{6}+49\beta b_{1}d_{7}+42\beta b_{2}d_{6}+35\beta b_{3}d_{5}+28\beta b_{4}d_{4}+21\beta b_{5}d_{3}\\ & +14\beta b_{6}d_{2}+7\beta b_{7}d_{1}+Gd_{5}+49b_{0}d_{7}+42b_{1}d_{6}+35b_{2}d_{5}+28b_{3}d_{4}+21b_{4}d_{3}\\ & +14b_{5}d_{2}+7b_{6}d_{1}) \end{array}$$

$$\begin{array}{ll} d_{9} & =-\frac{1}{72\beta b_{0}}(G\beta d_{7}+64\beta b_{1}d_{8}+56\beta b_{2}d_{7}+48\beta b_{3}d_{6}+40\beta b_{4}d_{5}+32\beta b_{5}d_{4}\\ & +24\beta b_{6}d_{3}+16\beta b_{7}d_{2}+8\beta b_{8}d_{1}+Gd_{6}+64b_{0}d_{8}+56b_{1}d_{7}+48b_{2}d_{6}+40b_{3}d_{5}\\ & +32b_{4}d_{4}+24b_{5}d_{3}+16b_{6}d_{2}+8b_{7}d_{1}) \end{array}$$

$$\begin{array}{ll} d_{10} & =-\frac{1}{90\beta b_{0}}(G\beta d_{8}+81\beta b_{1}d_{9}+72\beta b_{2}d_{8}+63\beta b_{3}d_{7}+54\beta b_{4}d_{6}+45\beta b_{5}d_{5}\\ & +36\beta b_{6}d_{4}+27\beta b_{7}d_{3}+18\beta b_{8}d_{2}+9\beta b_{9}d_{1}+Gd_{7}+81b_{0}d_{9}+72b_{1}d_{8}+63b_{2}d_{7}\\ & +54b_{3}d_{6}+45b_{4}d_{5}+36b_{5}d_{4}+27b_{6}d_{3}+18b_{7}d_{2}+9b_{8}d_{1}) \end{array}$$

$$\begin{array}{ll} d_{11} & =-\frac{1}{110\beta b_{0}}(G\beta d_{9}+100\beta b_{1}d_{10}+90\beta b_{2}d_{9}+80\beta b_{3}d_{8}+70\beta b_{4}d_{7}+60\beta b_{5}d_{6}\\ & +50\beta b_{6}d_{5}+40\beta b_{7}d_{4}+30\beta b_{8}d_{3}+20\beta b_{9}d_{2}+10\beta b_{10}d_{1}+Gd_{8}+100b_{0}d_{10}\\ & +90b_{1}d_{9}+80b_{2}d_{8}+70b_{3}d_{7}+60b_{4}d_{6}+50b_{5}d_{5}+40b_{6}d_{4}+30b_{7}d_{3}+20b_{8}d_{2}+10b_{9}d_{1}) \end{array}$$

$$\begin{array}{ll} d_{12} & =-\frac{1}{132\beta b_{0}}(G\beta d_{10}+121\beta b_{1}d_{11}+110\beta b_{2}d_{10}+99\beta b_{3}d_{9}+88\beta b_{4}d_{8}+77\beta b_{5}d_{7}\\ & +66\beta b_{6}d_{6}+55\beta b_{7}d_{5}+44\beta b_{8}d_{4}+33\beta b_{9}d_{3}+22\beta b_{10}d_{2}+11\beta b_{11}d_{1}+Gd_{9}\\ & +121b_{0}d_{11}+110b_{1}d_{10}+99b_{2}d_{9}+88b_{3}d_{8}+77b_{4}d_{7}+66b_{5}d_{6}+55b_{6}d_{5}+44b_{7}d_{4}\\ & +33b_{8}d_{3}+22b_{9}d_{2}+11b_{10}d_{1}) \end{array}$$

$$\begin{array}{ll} d_{13} & =-\frac{1}{156\beta b_{0}}(G\beta d_{11}+144\beta b_{1}d_{12}+132\beta b_{2}d_{11}+120\beta b_{3}d_{10}+108\beta b_{4}d_{9}+96\beta b_{5}d_{8}\\ & +84\beta b_{6}d_{7}+72\beta b_{7}d_{6}+60\beta b_{8}d_{5}+48\beta b_{9}d_{4}+36\beta b_{10}d_{3}+24\beta b_{11}d_{2}+12\beta b_{12}d_{1}+Gd_{10}\\ & +144b_{0}d_{12}+132b_{1}d_{11}+120b_{2}d_{10}+108b_{3}d_{9}+96b_{4}d_{8}+84b_{5}d_{7}+72b_{6}d_{6}+60b_{7}d_{5}+48b_{8}d_{4}\\ & +36b_{9}d_{3}+24b_{10}d_{2}+12b_{11}d_{1}) \end{array}$$

$$\begin{array}{ll} d_{14} & =-\frac{1}{182\beta b_{0}}(G\beta d_{12}+169\beta b_{1}d_{13}+156\beta b_{2}d_{12}+143\beta b_{3}d_{11}+130\beta b_{4}d_{10}+117\beta b_{5}d_{9}\\ & +104\beta b_{6}d_{8}+91\beta b_{7}d_{7}+78\beta b_{8}d_{6}+65\beta b_{9}d_{5}+52\beta b_{10}d_{4}+39\beta b_{11}d_{3}+26\beta b_{12}d_{2}+13\beta b_{13}d_{1}\\ & +Gd_{11}+169b_{0}d_{13}+156b_{1}d_{12}+143b_{2}d_{11}+130b_{3}d_{10}+117b_{4}d_{9}+104b_{5}d_{8}+91b_{6}d_{7}+78b_{7}d_{6}\\ & +65b_{8}d_{5}+52b_{9}d_{4}+39b_{10}d_{3}+26b_{11}d_{2}+13b_{12}d_{1}) \end{array}$$

$$\begin{array}{ll} d_{15} & =-\frac{1}{210\beta b_{0}}(G\beta d_{13}+196\beta b_{1}d_{14}+182\beta b_{2}d_{13}+168\beta b_{3}d_{12}+154\beta b_{4}d_{11}+140\beta b_{5}d_{10}\\ & +126\beta b_{6}d_{9}+112\beta b_{7}d_{8}+98\beta b_{8}d_{7}+84\beta b_{9}d_{6}+70\beta b_{10}d_{5}+56\beta b_{11}d_{4}+42\beta b_{12}d_{3}\\ & +28\beta b_{13}d_{2}+14\beta b_{14}d_{1}+Gd_{12}+196d_{14}b_{0}+182d_{13}b_{1}+168d_{12}b_{2}+154d_{11}b_{3}+140d_{10}b_{4}\\ & +126d_{9}b_{5}+112d_{8}b_{6}+98d_{7}b_{7}+84d_{6}b_{8}+70d_{5}b_{9}+56d_{4}b_{10}+42d_{3}b_{11}+28d_{2}b_{12}+14d_{1}b_{13}) \end{array}$$

$$\begin{array}{ll} d_{16} & =-\frac{1}{240\beta b_{0}}(75d_{5}b_{10}+Gd_{13}+210d_{14}b_{1}+120d_{8}b_{7}+105d_{7}b_{8}+195d_{13}b_{2}+225d_{15}b_{0}\\ & +135d_{9}b_{6}+30d_{2}b_{13}+60d_{4}b_{11}+45d_{3}b_{12}+150d_{10}b_{5}+180d_{12}b_{3}+165d_{11}b_{4}+90d_{6}b_{9}\\ & +15d_{1}b_{14}+135\beta b_{7}d_{9}+165\beta b_{5}d_{11}+150\beta b_{6}d_{10}+90\beta b_{10}d_{6}+G\beta d_{14}+120\beta b_{8}d_{8}\\ & +75\beta b_{11}d_{5}+60\beta b_{12}d_{4}+225\beta b_{1}d_{15}+15\beta b_{15}d_{1}+195\beta b_{3}d_{13}+30\beta b_{14}d_{2}+210\beta b_{2}d_{14}\\ & +45\beta b_{13}d_{3}+105\beta b_{9}d_{7}+180\beta b_{4}d_{12}) \end{array}$$
